# Phosphoinositide 3-kinase enables phagocytosis of large particles by terminating actin assembly through Rac/Cdc42 GTPase-activating proteins

**DOI:** 10.1038/ncomms9623

**Published:** 2015-10-14

**Authors:** Daniel Schlam, Richard D. Bagshaw, Spencer A. Freeman, Richard F. Collins, Tony Pawson, Gregory D. Fairn, Sergio Grinstein

**Affiliations:** 1 Division of Cell Biology, The Hospital for Sick Children, 555 University Avenue, Toronto, Ontario, Canada M5G1X8; 2 Institute of Medical Science, University of Toronto, Faculty of Medicine, 1 King's College Circle, Toronto, Ontario, Canada M5S1A8; 3 Lunenfeld-Tanenbaum Research Institute, Mount Sinai Hospital, 600 University Avenue, Toronto, Ontario, Canada M5G1X5; 4 Keenan Research Centre for Biomedical Science, St. Michael's Hospital, 209 Victoria Street, Toronto, Ontario, Canada M5B1T8

## Abstract

Phagocytosis is responsible for the elimination of particles of widely disparate sizes, from large fungi or effete cells to small bacteria. Though superficially similar, the molecular mechanisms involved differ: engulfment of large targets requires phosphoinositide 3-kinase (PI3K), while that of small ones does not. Here, we report that inactivation of Rac and Cdc42 at phagocytic cups is essential to complete internalization of large particles. Through a screen of 62 RhoGAP-family members, we demonstrate that ARHGAP12, ARHGAP25 and SH3BP1 are responsible for GTPase inactivation. Silencing these RhoGAPs impairs phagocytosis of large targets. The GAPs are recruited to large—but not small—phagocytic cups by products of PI3K, where they synergistically inactivate Rac and Cdc42. Remarkably, the prominent accumulation of phosphatidylinositol 3,4,5-trisphosphate characteristic of large-phagosome formation is less evident during phagocytosis of small targets, accounting for the contrasting RhoGAP distribution and the differential requirement for PI3K during phagocytosis of dissimilarly sized particles.

## Abstract (web summary)

Phagocytosis of large (but not small) particles requires PI 3-kinase activity. Here, Schlam *et al*. show that Rho GTPase-activating proteins are recruited to the phagocytic cup by products of PI 3-kinase, resulting in the local inactivation of Rac and Cdc42 and allowing for the completion of internalization of large particles.

## Graphical abstract

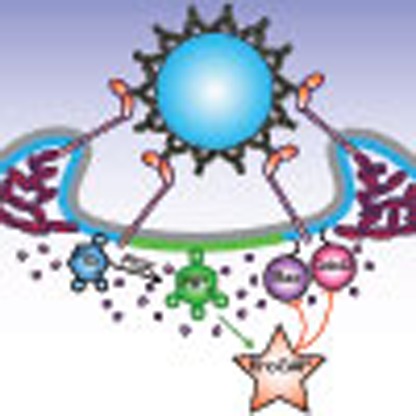

The elimination of microbial pathogens by phagocytosis is central to the innate immune response. Bacteria, fungi and other microorganisms are ingested and destroyed by professional phagocytes. Phagocytosis is also essential for the clearance of apoptotic cells, immune complexes and debris. Recognition of such disparate targets is enabled by the expression of a wide repertoire of phagocytic receptors on the surface of myeloid cells^1^. While sharing a similar overall outcome—the sequestration of targets in an intracellular vacuole termed the phagosome—the molecular events triggered by different types of phagocytic receptors vary considerably^2^. Moreover, emerging evidence suggests that large and small particles are engulfed by different mechanisms, even when the same receptor type is engaged. A striking example is provided by phosphatidylinositol 3,4,5-trisphosphate (PtdIns(3,4,5)P_3_): the phosphoinositide is required for the ingestion of large (⩾5 μm) particles but is dispensable for the uptake of smaller targets^3^,^4^.

A common feature of the phagocytic process—regardless of the type of receptor bound or the size of the target particle—is the involvement of the actin cytoskeleton^5^. In all instances, actin-rich protrusions facilitate the initial capture of phagocytic targets and the extension of pseudopods that ultimately surround the particle, promoting fission of the sealed vacuole^6^. Rho-family GTPases orchestrate the remodelling of actin during phagocytosis^7^,^8^,^9^,^10^,^11^. Of note, the progression of pseudopods and the completion of phagocytosis involve not only actin polymerization but also its subsequent disassembly, particularly in the case of larger particles. The sites where targets are initially engaged and actin first assembles begin to dismantle even as the pseudopods continue to progress along the particle's surface. It is unclear whether this coordinated disassembly is required to recycle limiting components to the tips of the advancing pseudopods, enables membrane deformation and/or clears a path for focal exocytosis of endomembranes. Notably, most of the available information regarding the cytoskeletal remodelling that accompanies phagocytosis pertains to the initial stages of F-actin polymerization, while much less is known about the mechanisms of termination and disassembly. Here, we show that actin breakdown is critical for the uptake of large particles and describe a phosphoinositide 3-kinase (PI3K)-driven mechanism whereby Rho GTPase activity and actin polymerization are acutely terminated, enabling completion of phagocytosis. Our observations account for the differential requirement for PtdIns(3,4,5)P_3_ biosynthesis during phagocytosis of small versus large particles.

## Results

### PI3K directs F-actin disassembly at large phagocytic cups

**Figure 1:**
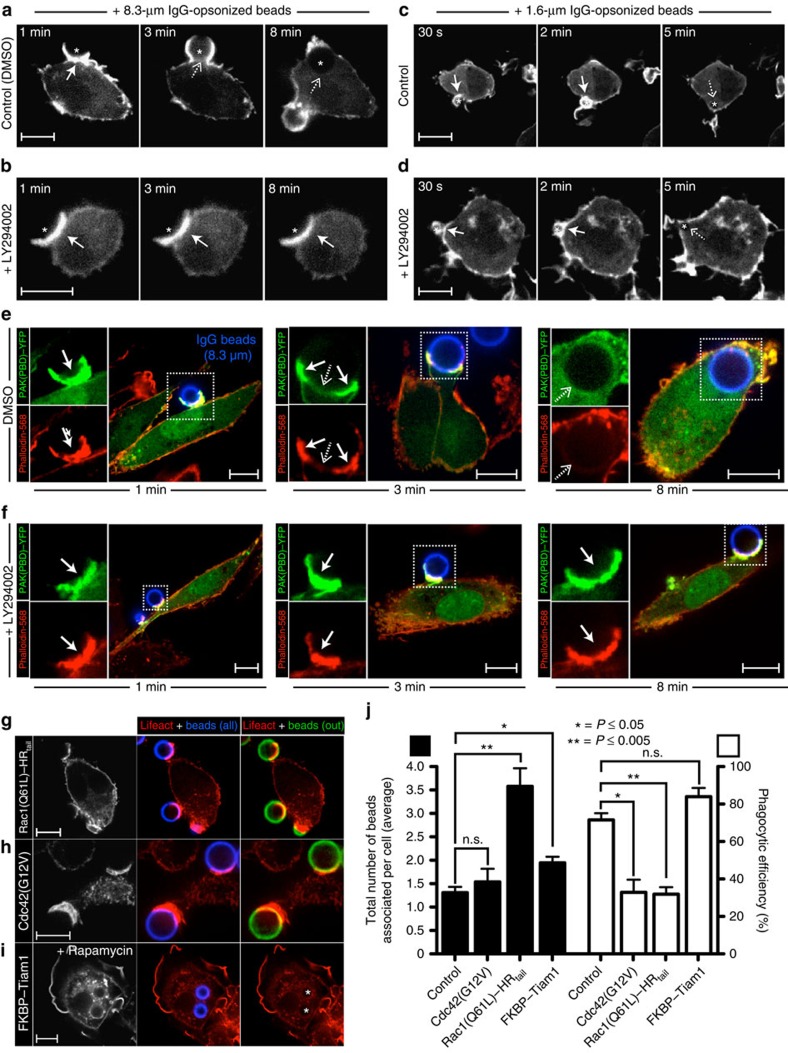
PI3K controls actin disassembly during phagocytosis of large targets. (**a**–**d**) Time-lapse confocal micrographs of RAW 264.7 macrophages transiently expressing Lifeact–mRFP and challenged with 8.3-μm (**a**,**b**) or 1.6-μm (**c**,**d**) IgG-opsonized beads (signalled with a star). Cells were treated with vehicle (DMSO) or the PI3K inhibitor LY294002 for 10 min before initiating phagocytosis. Actin dynamics were followed throughout the course of engulfment, with the 0-min time point corresponding to the initial engagement of the beads. Solid and dashed arrows point to sites of F-actin accumulation and clearance, respectively. (**e**,**f**) Confocal micrographs of primary human macrophages transfected with PAK(PBD)–YFP, a biosensor for active Rac/Cdc42, during phagocytosis of 8.3 μm IgG beads. Before phagocytosis, cells were treated with DMSO (**e**) or LY294002 (**f**) for 10 min. Phagocytosis was allowed to proceed for the indicated times, before fixing and staining F-actin with phalloidin. Insets (boxed regions) show magnified views of the phagocytic cup. (**g**–**i**) Confocal micrographs of RAW 264.7 macrophages transiently co-expressing Lifeact–mRFP in combination with constitutively active Rac1 (**g**), constitutively active Cdc42 (**h**) or a recruitable form of the Rac1 GEF Tiam1 (**i**). Addition of rapamycin triggers translocation of Tiam1 to the plasmalemma, where its rapamycin-binding domain (FKBP) interacts with a second, complementary rapamycin-binding moiety. Transfectants were challenged with 8.3-μm IgG beads, and phagocytosis allowed to proceed for 10 min before fixation. All phagocytic targets were stained with Cy5-conjugated secondary antibody (shown in blue) before phagocytosis. Extracellular beads were identified by staining fixed (non-permeabilized) cells with an Alexa Fluor 488-conjugated secondary antibody (shown in green). Internalized beads are indicated with a star. Scale bar, 10 μm. (**j**) Quantification of phagocytic indices (total number of beads associated per cell; black bars) and phagocytic efficiencies (ratio of internalized-to-total number of beads per cell; white bars) for the experiments described in (**g**–**i**). Values represent the means of three independent replicates±s.e.m. At least 25 cells were assessed per replicate. *P≤0.05, **P≤0.005 or n.s. (not significant) using Student's two-tailed unpaired *t*-tests.

In cells treated with PI3K inhibitors, the initial stages of phagocytosis are seemingly normal. However, if the particles are large, the engulfment process cannot be completed. We questioned whether aberrant cytoskeletal dynamics could account for the inability of cells with impaired PI3K activity to finalize uptake of large targets. To test this experimentally, we visualized F-actin in macrophages during the course of phagocytosis in the presence and absence of the PI3K inhibitor LY294002. RAW 264.7 macrophages transfected with Lifeact–monomeric red fluorescent protein (mRFP; fluorescent reporter of filamentous actin^12^) were incubated with vehicle (dimethylsulphoxide (DMSO)) alone or LY294002 before being challenged with either large (8.3 μm) or small (1.6 μm) IgG-coated particles to engage Fcγ receptors. As illustrated in Fig. 1a, F-actin accumulated markedly at developing cups during the early stages (≈1 min) of large-particle ingestion. Of note, the progression of pseudopods along the perimeter of the particle was accompanied by the disassembly of F-actin from the base of the cup (3 min panel; dashed arrow) and, once phagosomes had sealed, actin was only detectable at the site of scission (8 min panel; see Supplementary Movie 1 for uninterrupted time-lapse sequence). The early phase of actin assembly was also observed in macrophages treated with LY294002 (Fig. 1b; solid arrow). However, as reported earlier^3^, the extension of pseudopods was arrested, yielding shallow abortive cups where polymerized actin persisted for extended periods (see 3 and 8 min panels in Fig. 1b and also Supplementary Movie 2). These results suggest that PI3K is dispensable for the initial polymerization of actin that drives membrane deformation, but is critical for the secondary actin clearance that accompanies internalization of large particles.

Phagocytes challenged with small (1.6 μm) beads similarly accumulated actin at nascent cups (Fig. 1c; 30 s panel). However, the beads quickly became entirely surrounded by F-actin (2 min panel), and sealed before disassembly at the base of the cup was apparent (5 min panel). Interestingly, when ingesting small beads, actin dynamics in cells treated with LY294002 (Fig. 1d) were similar to those in vehicle-treated controls, and phagocytosis proceeded to completion, as reported^3^. The differential effect of LY294002 on actin dynamics and on the phagocytic capacity of cells challenged with small versus large particles suggested that PI3K-dependent actin disassembly may be required for the uptake of large, but not small targets.

### Rac/Cdc42 inactivation accompanies completion of phagocytosis

Because the Rho GTPases Rac and Cdc42 are key to the assembly of filamentous actin during FcγR-mediated phagocytosis^8^,^13^,^14^,^15^, we speculated that their inactivation would facilitate actin disassembly, and that this was mediated by PI3K. We assessed this experimentally in primary macrophages using PAK(PBD)–yellow fluorescent protein (YFP), a fluorescent biosensor of Rac/Cdc42 activity consisting of the p21-binding domain (PBD) of p21-activated kinase (PAK)^9^,^16^. Human monocyte-derived macrophages transiently expressing PAK(PBD)–YFP were challenged with 8.3-μm IgG-opsonized beads. Phagocytosis was then allowed to proceed for 1, 3 or 8 min before fixation and staining of F-actin with phalloidin-568 (Fig. 1e,f). As expected, sites of particle engagement displayed prominent PAK(PBD)–YFP enrichment (corresponding to sites of GTP-bound Rac and/or Cdc42) and colocalized with sites of intense F-actin staining (1 min time point in Fig. 1e; solid arrow). Importantly, in otherwise untreated cells disassembly of F-actin from the base of the cup was coincident in space and time with the displacement of the active Rac/Cdc42 biosensor (3 min time point; dashed arrow), implying that inactivation of these small G proteins accompanied F-actin breakdown. Fully internalized phagosomes lacked both actin and PAK(PBD)–YFP (8 min time point; dashed arrow). In sharp contrast, cells treated with LY294002 displayed sustained Rac/Cdc42 activity at the base of an actin-rich abortive cup, as indicated by the retention of the PAK(PBD)–YFP biosensor and by the intense phalloidin staining (1–8 min time points in Fig. 1f; solid arrow). An identical dependency of actin disassembly and Rac/Cdc42 inactivation on PI3K activity was also observed in RAW 264.7 macrophages (Supplementary Fig. 1). Together, these results are consistent with the notion that PI3K signals the termination of Rac and/or Cdc42 activity, which in turn is necessary for F-actin disassembly and phagocytosis of large particles.

### Role of RhoGEF inactivation in phagocytosis completion

Two distinct mechanisms could account for the sustained Rac and/or Cdc42 signalling observed at abortive phagocytic cups in LY294002-treated cells: prolongation of the activity of guanine nucleotide exchange factors (GEFs), or impairment of GTPase-activating proteins (GAPs). To differentiate between these possibilities we forced the association of Tiam1, a Rac1 GEF^17^, with the plasmalemma by means of a rapamycin-induced heterodimerization system^18^ (see Methods). We reasoned that if GEF displacement was indeed necessary for Rac1 inactivation, then the sustained association of this recombinant GEF with the membrane should lead to persistent Rac1 signalling and formation of abortive, F-actin-rich phagocytic cups akin to those shown in Fig. 1b,f. RAW 264.7 cells transiently expressing the heterodimerization system and Lifeact–mRFP were acutely exposed to rapamycin, triggering translocation of Tiam1 to the plasmalemma (Supplementary Movie 3; Fig. 1i). Note the extensive membrane ruffling induced by Tiam1 mobilization. Sustained recruitment of the GEF did not, however, prevent the effective uptake of large particles, which were fully internalized as indicated by their inaccessibility to externally added secondary antibodies (Fig. 1i,j). These results suggest that the displacement of RhoGEFs from the nascent phagocytic is not a necessary condition for Rho GTPase inactivation or for F-actin disassembly. Nonetheless, we cannot rule out the possibility that removal or inactivation of GEFs occurs during the normal progression of phagocytosis.

In contrast to the inability of FKBP–Tiam1 to arrest phagocytosis, uptake of IgG-coated beads was markedly reduced in macrophages transiently expressing either Rac1(Q61L)–HR_tail_ or Cdc42(G12V), constitutively active forms of the GTPases (Fig. 1g,h,j). Of note, the number of particles associated with the cells in fact increased in Rac1(Q61L)–HR_tail_ transfectants, suggesting that internalization, as opposed to binding, was impaired. Accordingly, formation of nascent cups with considerable accumulation of F-actin was evident in these cells (Fig. 1g). Similar abortive cups subtended by rich actin networks were observed in Cdc42(G12V) transfectants (Fig. 1h), as previously described^19^. Together, these results confirm that Rac/Cdc42 inactivation is necessary for actin breakdown and completion of phagocytosis, and suggest that recruitment and/or activation of RhoGAPs—as opposed to displacement of RhoGEFs—is required to turn off the GTPases. This notion is bolstered by the observation that, while Tiam1 remained associated with internalized phagosomes, no F-actin was detected on their membranes (Fig. 1i).

### Subcellular distribution of RhoGAPs during phagocytosis

**Figure 2:**
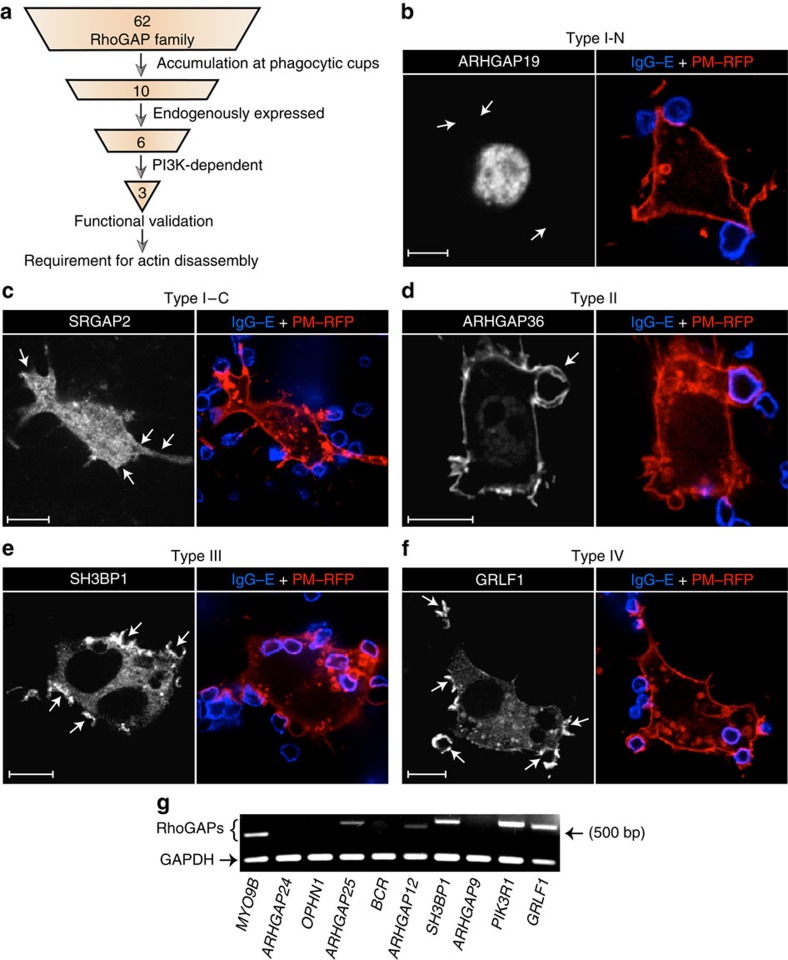
Screening for RhoGAPs responsible for orchestrating actin breakdown. (**a**) Schematic outlining the experimental approach used in this study. We generated a collection of constructs comprising 62 members of the RhoGAP family conjugated to mCitrine. Each plasmid was independently transfected into RAW 264.7 macrophages, and the subcellular distribution of the encoded RhoGAP followed by confocal microscopy during phagocytosis. The extent of accumulation at phagocytic cups was assessed by ratiometric analysis relative to PM–RFP, a marker for bulk plasma membrane. The 10 RhoGAPs that most significantly accumulated at phagocytic cups were selected for further screening, consisting of assessing endogenous expression by RT–PCR and dependency of recruitment on PI3K. The ability of the remaining candidates to function as RhoGAPs during phagocytosis was validated by overexpressing these proteins and quantifying phagocytic capacity. Lastly, gene silencing was used to determine whether the identified RhoGAPs were necessary for coordinating actin remodelling during phagocytosis. (**b**–**f**) Subcellular distribution of a selective subset of RhoGAPs during phagocytosis of IgG-coated erythrocytes (IgG–E). RAW 264.7 macrophages were co-transfected with constructs encoding mCitrine-tagged RhoGAPs plus the plasmalemmal marker PM–RFP. Each panel corresponds to a representative member of four identifiable RhoGAP types, as determined by their recruitment (or lack thereof) to sites of phagocytosis. RhoGAPs that failed to localize to sites of particle engagement and remained nuclear (**b**) or cytosolic (**c**) were assigned a type I–N and type I–C nomenclature, respectively. RhoGAPs that constitutively localized to the plasmalemma but did not accumulate at phagocytic cups (**d**) were designated as type II. In contrast, RhoGAPs that were initially cytosolic but translocated to phagocytic cups (**e**) were labelled as type III. Lastly, type IV RhoGAPs showed both a cytosolic and plasmalemmal distribution at rest but accumulated at phagocytic cups (**f**). Arrows point to sites of particle engagement. Scale bar, 10 μm. At least 20 cells were assessed for each RhoGAP in the collection. (**g**) Representative image of three independent RT–PCR assays, used to ascertain the endogenous expression levels of the 10 RhoGAPs that were most markedly recruited to phagocytic cups. GAPDH was used as a positive control.

The human genome encodes more than 60 members of the RhoGAP family^20^. Identification of the putative RhoGAP(s) involved in phagocytosis is further compounded by the fact that the GTPase(s) targeted by each of the RhoGAPs are, in many cases, not known. To overcome these problems, we conducted an unbiased screen of the RhoGAP family, using the strategy outlined in Fig. 2a. As an initial selection criterion, we assessed which RhoGAPs translocated to sites of phagocytosis. To this end, we generated fluorescent chimeras of the 62 available RhoGAPs, individually transfected them into macrophages, and assessed their presence at phagocytic cups induced by exposure to IgG-coated particles. The tagged constructs were co-transfected with palmitoylated-myristoylated RFP (PM–RFP), a fluorescent plasmalemmal marker that enabled us to normalize the accumulation of the RhoGAPs per unit membrane area. Confocal images were acquired 3 min after the macrophages were exposed to the particles, and ratios of the fluorescence of the two probes were calculated. These ratios were then used to score the degree of RhoGAP recruitment to the cup (a relative scale ranging from a minimum of 0 to a maximum of 6 was used). Supplementary Fig. 2 includes representative micrographs of the cellular distribution of all 62 RhoGAPs and provides their corresponding recruitment score. A subset of representative images are also shown in Fig. 2b–f, where four types of distribution are differentiated: RhoGAPs that failed to translocate to the phagocytic cup were classified either as type I–N or type I–C, depending on whether they remained in nuclear or cytosolic compartments, respectively (Fig. 2b,c). RhoGAPs that were present at the plasmalemma before particle engagement but did not accumulate at the phagocytic cup were designated as type II (Fig. 2d). Of most interest were types III and IV, which were either cytosolic or both membrane-associated and cytosolic at rest, respectively, but accumulated prominently at phagocytic cups (Fig. 2e,f). Ten different RhoGAPs were found to clearly accumulate (score ⩾4) at the phagocytic cup.

Because our screening process involved ectopic expression of tagged proteins, it was imperative to verify whether the candidate RhoGAPs were endogenously expressed in macrophages. As shown in Fig. 2g, we used reverse transcription polymerase chain reaction (RT–PCR) to validate their expression. Following 19 cycles of amplification, this analysis revealed that only 6 of the 10 candidate GAPs (*MYO9B*, *ARHGAP25*, *ARHGAP12*, *SH3BP1*, *PIK3R1* and *GRLF1*) were present at moderate-to-high levels in RAW 264.7 macrophages, as judged by the level of mRNA expression (Fig. 2g).

### PI3K-dependent translocation of RhoGAPs to phagocytic cups

**Figure 3:**
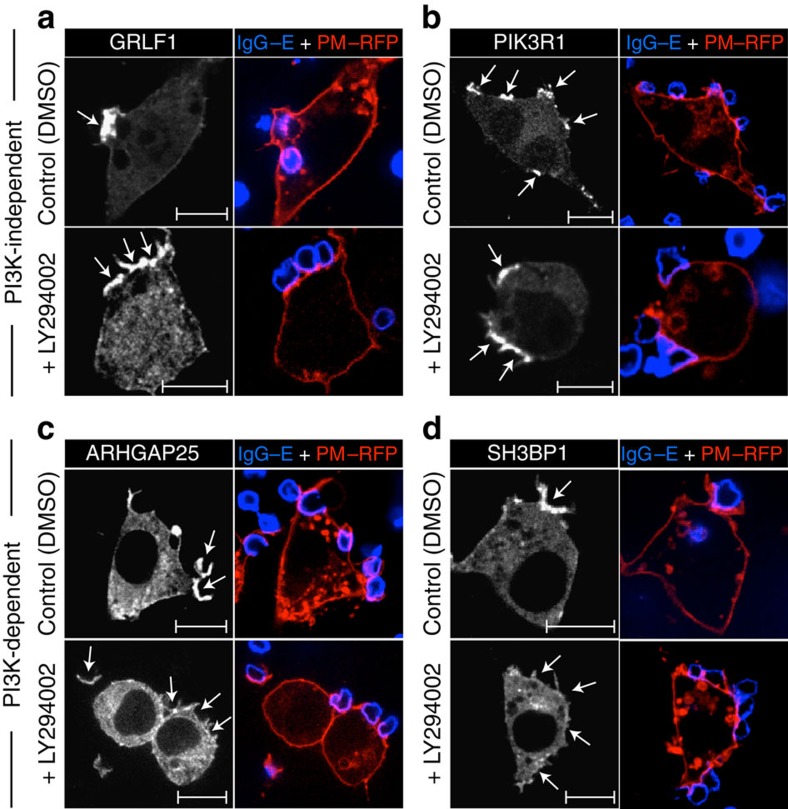
PI3K dependency of RhoGAP recruitment to phagocytic cups. (**a**–**d**) RAW 264.7 macrophages were co-transfected with constructs encoding each of the 10 RhoGAPs that most markedly accumulated at phagocytic cups as well as with the plasmalemmal marker PM–RFP. Transfectants were exposed to IgG-opsonized erythrocytes (IgG–E; shown in blue) for 3 min before being fixed and imaged by confocal microscopy. Where indicated, cells were treated with either vehicle (DMSO) or the PI3K inhibitor LY294002 for 10 min before the addition of phagocytic targets. Only a subset of the 10 RhoGAPs that were investigated is shown, namely two PI3K-independent (**a**,**b**) and two PI3K-dependent (**c**,**d**) proteins. (see Supplementary Fig. 3 for the remaining RhoGAPs that were investigated.) Arrows point to sites of particle engagement. Scale bar, 10 μm. Micrographs are representative of three independent experiments. At least 20 cells were assessed per replicate for each of the RhoGAPs examined.

Given that PI3K inhibition resulted in the formation of abortive phagocytic cups displaying prolonged Rac and Cdc42 activity (Fig. 1f; Supplementary Fig. 1b), we considered the possibility that Rho-family GAPs were recruited to sites of phagocytosis by products of PI3K. To test this hypothesis, we transfected RAW 264.7 cells with plasmids encoding those RhoGAPs found to be recruited to the phagocytic cup. Transfectants were treated with vehicle or LY294002 for 10 min before being challenged with IgG-coated erythrocytes and imaged by confocal microscopy after fixation (Fig. 3a–d). For brevity, representative images of only two PI3K-sensitive and two PI3K-insensitive RhoGAPs are shown in Fig. 3; the remaining images are included in Supplementary Fig. 3. The collated data, summarized in Table 1, revealed that only three of the GAPs endogenously expressed in macrophages—ARHGAP12, ARHGAP25 and SH3BP1—require PI3K activity to associate with the phagocytic cup. It is noteworthy that ARHGAP12, ARHGAP25 and SH3BP1 are known to exert their catalytic function on Rac and/or Cdc42 (refs 21, 22, 23).

To assess whether these RhoGAPs behave similarly in primary cells, human monocyte-derived macrophages transiently expressing mCitrine-tagged ARHGAP12, ARHGAP25 or SH3BP1 were challenged with IgG-coated erythrocytes in the presence or absence of a PI3K inhibitor. As expected, all three GAPs noticeably accumulated at developing phagocytic cups in control conditions, but their recruitment was precluded in the presence of LY294002 (Supplementary Fig. 4).

### Validation of RhoGAP function during phagocytosis

**Figure 4:**
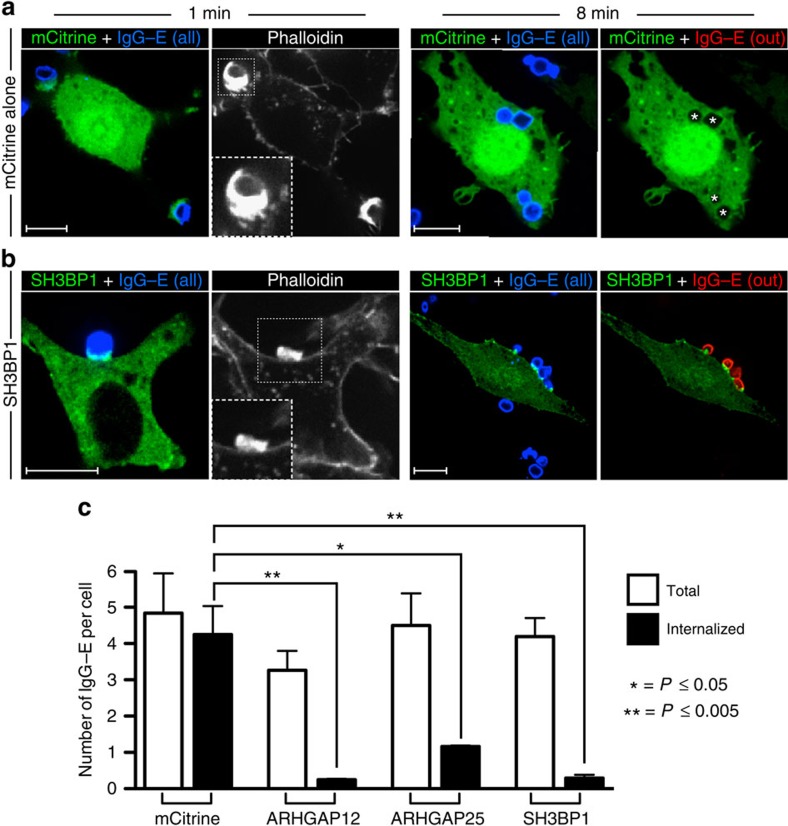
Functional validation of candidate RhoGAPs. (**a**,**b**) Constructs encoding mCitrine alone (**a**) or SH3BP1 (**b**) were electroporated into RAW 264.7 macrophages to yield high levels of expression. To determine whether these RhoGAPs could indeed inactivate GTPases that are instrumental for phagocytosis, electroporated cells were challenged with IgG-opsonized erythrocytes and allowed to interact with the targets for 1 min (left panels) or 8 min (right panels) before fixation. All phagocytic targets (shown in blue) were stained with a Cy5-conjugated anti-IgG secondary antibody before addition to phagocytes. To determine the number of IgG-coated erythrocytes that were not internalized, fixed (non-permeabilized) cells were stained with Cy3-conjugated anti-IgG (shown in red). Cells were then washed, permeabilized and stained for F-actin with phalloidin. Insets show magnified views of nascent phagocytic cups (boxed region) at the 1 min time point. Phagosomes that had already been formed before addition of the Cy3-conjugated secondary antibody are indicated with a star. Scale bar, 10 μm. Micrographs are representative of three independent experiments. (**c**) Quantification of the total number of IgG-coated erythrocytes that were engaged (white bars) or internalized (black bars) per phagocyte overexpressing mCitrine alone or mCitrine-tagged ARHGAP12, ARHGAP25 or SH3BP1. Values in (**c**) represent the means of three independent replicates±s.e.m. *P≤0.05 and **P≤0.005 using Student's two-tailed unpaired *t*-tests. At least 20 cells were assessed per replicate for each of the RhoGAPs examined.

Having identified ARHGAP12, ARHGAP25 and SH3BP1 through the screening process described above, we examined their functional activity in phagocytes. We hypothesized that if these RhoGAPs were indeed capable of inactivating Rac and/or Cdc42, their overexpression would hinder Rho GTPase-mediated actin nucleation and thus impair phagocytosis at an early stage. We tested this premise using electroporation, which enabled us to express high levels of mCitrine-tagged ARHGAP12, ARHGAP25 or SH3BP1 in the macrophages. Unconjugated mCitrine was used as a control to rule out untoward effects of the electroporation procedure. As illustrated in Fig. 4, deliberate overexpression of the mCitrine chimeras of ARHGAP12, ARHGAP25 or SH3BP1 markedly depressed the phagocytic efficiency of RAW 264.7 cells, compared with unconjugated mCitrine. It is noteworthy that this phagocytic impairment was accompanied by reduced F-actin accumulation at sites of contact with target particles (Fig. 4b), where only modest pedestals were formed instead of the elaborate phagocytic cups normally observed. These observations support the notion that ARHGAP12, ARHGAP25 and SH3BP1 can function as RhoGAPs in macrophages during phagocytosis.

That RhoGAP overexpression impairs phagocytic capacity may seem at odds with a requirement for Rac and Cdc42 inactivation for efficient engulfment. However, these observations can be readily reconciled given that both the initial activation and subsequent inactivation of these Rho GTPases are critical for the orderly extension and progression of pseudopodia. Indeed, the immature membrane pedestals that form at sites of phagocytosis in ARHGAP12, ARHGAP25 or SH3BP1 transfectants suggest that the overexpressed RhoGAPs terminate Rac and Cdc42 signalling prematurely. Thus, cycling of the GTPases between active and inactive states is instrumental for successful phagocytosis of large particles, as both premature termination of Rho GTPase activity and protracted signalling by these proteins are deleterious to the completion of the phagocytic response.

### RhoGAPs cooperate in F-actin breakdown at phagocytic cups

**Figure 5:**
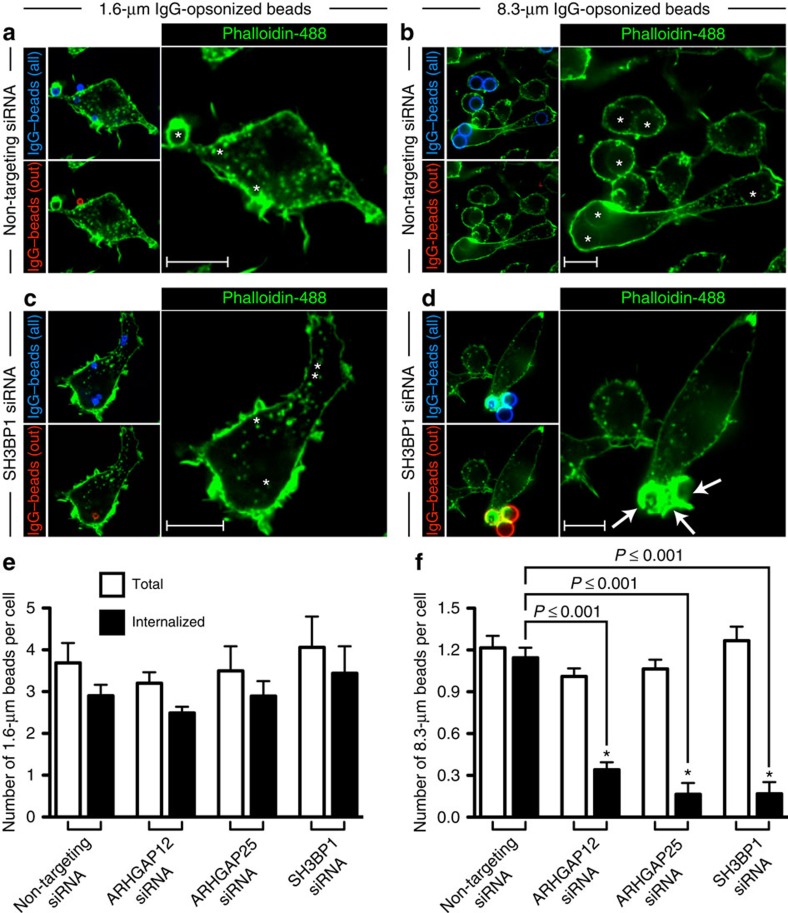
RhoGAPs are necessary for actin breakdown and uptake of large targets. (**a**–**d**) RAW 264.7 macrophages were electroporated twice (sequentially within a 48-h interval) with non-targeting control siRNA (**a**,**b**) or with siRNA directed against *SH3BP1* (**c**,**d**), *ARHGAP12* or *ARHGAP25* and challenged with small (left panel) or large (right panel) IgG-coated beads 96 h after the initial electroporation. Cells were allowed to phagocytose for 10 min before fixation. All phagocytic targets (shown in blue) were stained with Cy5-conjugated anti-IgG secondary antibody before being added to phagocytes, and particles that remained extracellular (shown in red) were identified as in Fig. 4. Cells were then washed, permeabilized and stained for F-actin with phalloidin. Formed phagosomes are indicated with a star. Arrows point to abortive, actin-rich phagocytic cups that develop as a result of RhoGAP silencing in cells attempting to engulf large targets. Scale bar, 10 μm. (**e**,**f**) Quantification of the experiment described in (**a**–**d**). The total number of IgG-coated beads that were engaged (white bars) or internalized (black bars) per phagocyte is plotted. Values in (**e**,**f**) represent the means of three independent replicates±s.e.m. *P≤0.001 using Student's two-tailed unpaired *t*-tests. At least 20 cells were assessed per replicate for each of the siRNAs examined.

We used RNA interference to assess the role of the PI3K-sensitive GAPs in actin disassembly during phagocytosis of both small and large particles. RAW 264.7 macrophages were electroporated with non-targeting control small interfering RNA (siRNA) or with siRNA directed against *ARHGAP12*, *ARHGAP25* or *SH3BP1*. Knockdown efficiency was assessed by immunoblotting, which validated the effectiveness and specificity of the silencing strategy; the oligonucleotides designed to silence each one of the three RhoGAPs markedly reduced the expression of the intended target, while the protein levels of the other two GAPs remained unaffected (Supplementary Fig. 5a–c). To evaluate the functional contribution of each one of the GAPs, the siRNA-treated cells were challenged with small (1.6 μm) or large (8.3 μm) IgG-coated beads, and phagocytosis was allowed to proceed for 10 min before fixation. Targets that remained extracellular were then identified by addition of a Cy3-conjugated anti-IgG antibody. As shown in Fig. 5a,b, electroporation with non-targeting siRNA had no discernible effect on the phagocytosis of either small or large targets, as these cells internalized the majority of bound particles. In contrast, phagocytosis of large particles was markedly depressed in cells in which *SH3BP1* was silenced (Fig. 5d,f). Such cells developed abortive, actin-dense phagocytic cups (indicated with an arrow in Fig. 5d) that extended only partway around the large phagocytic targets. Of note, phagocytosis of small beads was not significantly affected in these cells (Fig. 5c,e). A similar pattern was observed in cells electroporated with siRNA directed against *ARHGAP12* and *ARHGAP25*; phagocytosis of large beads was inhibited to varying extents, but that of small beads was only marginally affected (see Supplementary Fig. 5d–g for representative micrographs). Quantification of the results of three independent phagocytosis experiments is provided in Fig. 5e,f.

### Dependency of PI3K signalling on phagocytic-target size

**Figure 6:**
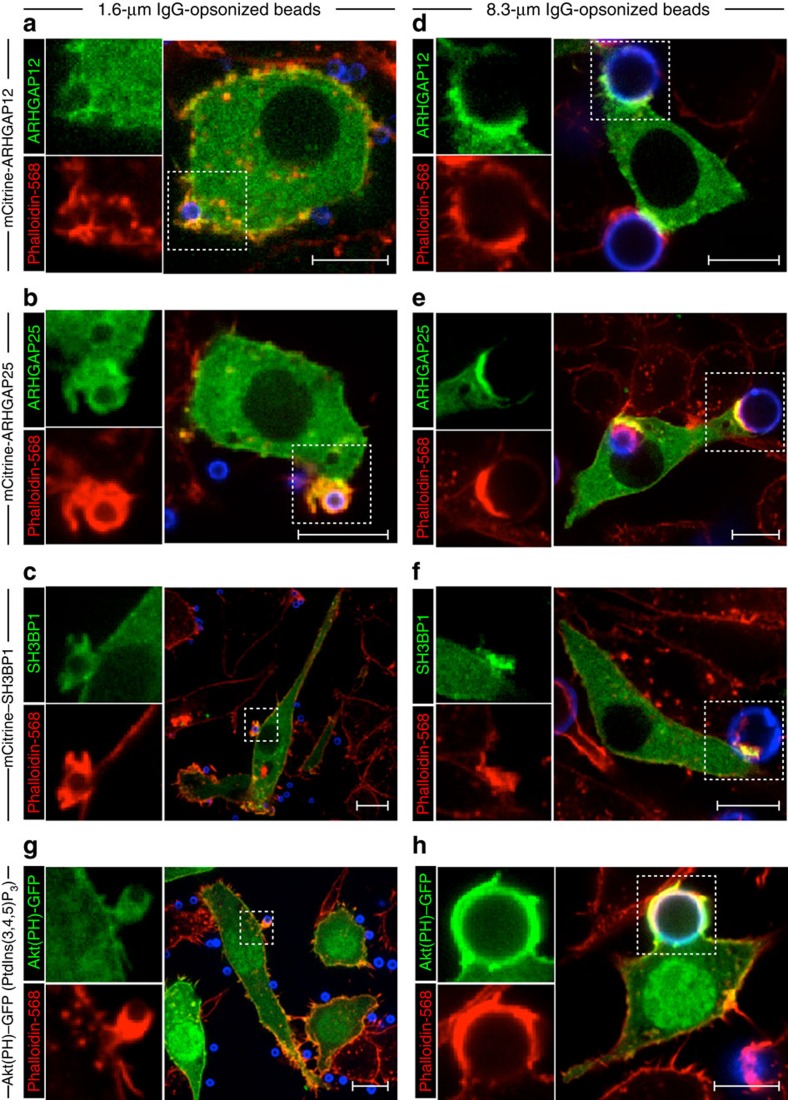
Role of PtdIns(3,4,5)P_3_ and RhoGAPs during uptake of small particles. Representative confocal micrographs of RAW 264.7 macrophages transfected with constructs encoding mCitrine-tagged ARHGAP12 (**a**,**d**), ARHGAP25 (**b**,**e**), SH3BP1 (**c**,**f**) or with GFP-tagged Akt(PH), a probe for PtdIns(3,4,5)P_3_ (**g**,**h**). The distribution of these fluorescent proteins is shown in green. Cells were challenged with either small (1.6 μm; left panels) or large (8.3 μm; right panels) IgG-coated beads to initiate phagocytosis. All beads were stained with a Cy5-conjugated secondary antibody (shown in blue) before being sedimented onto macrophage surfaces. Phagocytosis was allowed to continue for 8 min before fixation and staining of F-actin with phalloidin-568 (shown in red). Insets (boxed regions) show magnified views of the phagocytic cup. Scale bar, 10 μm. Micrographs are representative of three independent experiments; at least 20 cells were assessed per replicate for each condition.

Given that expression of ARHGAP12, ARHGAP25 and SH3BP1 was required for the uptake of large particles but dispensable for that of smaller ones, it was conceivable that these GAPs are recruited to large, but not small nascent phagosomes. To address this possibility, RAW 264.7 macrophages transiently expressing fluorescent chimeras of the three RhoGAPs were challenged with either small (Fig. 6a–c) or large (Fig. 6d–f) IgG-coated beads. As expected, all three GAPs were robustly recruited to sites of large-particle engagement. Of interest, however, their recruitment was inconspicuous when the small particles were used. The relative absence of these RhoGAPs is consistent with the observation that they are dispensable for phagocytosis of small particles.

Since these critical RhoGAPs mobilize to large phagocytic cups in a PI3K-dependent fashion, we questioned whether their inability to translocate to small cups was attributed to diminished PtdIns(3,4,5)_3_ formation when using small particles as targets. To assess this, PtdIns(3,4,5)_3_ distribution was monitored using Akt(PH)-GFP, a fluorescent biosensor consisting of the pleckstrin homology domain of Akt fused to green fluorescent protein. Strikingly, PtdIns(3,4,5)_3_ formation was not readily apparent in response to small phagocytic targets (Fig. 6g), contrasting with its much more prominent accumulation in large phagocytic cups (Fig. 6h). Together, these observations suggest that PI3K-dependent recruitment of RhoGAPs is necessary for F-actin disassembly during the internalization of large particles. Our observations also account, at least in part, for the differential requirement for PI3K in the phagocytosis of dissimilarly sized particles.

## Discussion

Rapid and extensive dismantling of actin networks is routinely observed during or immediately after phagosome sealing. Removal of cortical actin is thought to enable the fusion and fission events underlying phagosome maturation^24^. In addition, in the case of large particles, F-actin breakdown appears to be necessary for successful engulfment. Three alternative mechanisms could explain the need for F-actin disassembly: persistence of the cortical cytoskeleton may limit the ability of the forming phagosomal membrane to curve around the target. We regard this possibility as unlikely, inasmuch as phagocytosis of small particles—which exhibit a higher degree of curvature than large ones—is less dependent on F-actin disassembly. A second possibility is that internalization of large particles requires significant expansion of the lining membrane. Expansion may result from elastic stretching of the pre-existing membrane or, more likely, from exocytic insertion of endomembranes at sites of phagocytosis^25^,^26^,^27^,^28^. Both of these processes would be impeded if the underlying cytoskeleton persisted. A third and in our view more plausible alternative is that as actin polymerizes during the formation of large phagocytic cups, one or more determinants of the assembly process may be exhausted, limiting the rate of growth of advancing pseudopodia. Replenishment would only occur following disassembly and recycling of the components initially engaged at the base of the phagocytic cup. Regardless of the underlying purpose, it is clear that F-actin disassembly is essential to complete the internalization of large targets—such as fungi or apoptotic bodies—and is accompanied by inactivation of the Rho-family GTPases that initiate the polymerization event.

The notion that completing internalization of large endocytic vacuoles is contingent on the inactivation of the Rho GTPases responsible for initiating actin polymerization is consistent with a recent study making use of a photoactivatable form of Rac1; nascent macropinocytic cups rapidly developed on Rac1 photoactivation at the plasmalemma, but sealed only once Rac1 activation had been reversed by interrupting illumination^29^.

Our data indicate that recruitment and/or stimulation of Rho-family GAPs is critical for the disassembly of actin when large particles are ingested. Moreover, they establish a link between the synthesis of 3′-phosphoinositides and the downregulation of Rho GTPase activity. These observations are in good agreement with the findings of Swanson and colleagues^4^, who originally identified PI3K as an essential component of the phagocytic machinery and subsequently reported that inhibition of the kinase leads to sustained GTPase signalling in stalled phagocytic cups^19^. They are also consistent with the observation of Cox *et al*.^3^ that phagocytosis of large, but not small particles is impaired by wortmannin, a PI3K antagonist. The present work extends and clarifies these observations by identifying active GAPs recruited to the phagocytic membrane by 3′-phosphoinositides.

It is noteworthy that not one, but three separate RhoGAPs—SH3BP1, ARHGAP12 and ARHGAP25—are required for completion of phagocytosis. This reflects, in part, the fact that multiple Rho-family GTPases are activated during phagocytosis^7^,^13^,^30^. It also suggests that the complex deactivation process is dictated by the asymmetric nature of the distribution and activation of the Rho GTPases: Rac is activated primarily at the base of the cup while Cdc42 is active at the tips of pseudopodia, fostering membrane extension^9^. In this regard, it is important to note that SH3BP1, ARHGAP12 and ARHGAP25 display varying degrees of GAP activity towards Rac or Cdc42 (refs 21, 22, 23, 31). As such, one would expect them to be differentially effective in different subdomains of the nascent phagosome, contributing to the asymmetric and asynchronous pattern of activation and deactivation of the GTPases.

The observation that sustained signalling by either Rac or Cdc42 can compromise phagocytic efficiency may help explain why multiple GAPs need to be engaged and why their effects are not redundant, but rather synergistic. For instance, silencing one of the GAPs may result in prolonged Cdc42 activity—even if Rac is inactivated—which would be sufficient to hinder phagocytosis (Fig. 5f). Thus, despite their functional similarities, SH3BP1, ARHGAP12 and ARHGAP25 are all required and act synergistically to disassemble actin in a timely and spatially coordinated manner.

We have shown that FcγR-mediated phagocytosis is aborted at a relatively early stage when any one of the three RhoGAPs is overexpressed in macrophages (Fig. 4). These findings are in agreement with a previous study reporting that phagocytosis of serum-opsonized yeast is impaired in engineered phagocytes (COS^phox^FcγR cells) overexpressing ARHGAP25 (ref. 23). However, that study also reported that silencing ARHGAP25 in PLB cells and in human macrophages *increases* phagocytosis of serum-opsonized particles, implicating ARHGAP25 as a negative regulator of phagocytosis. This observation seems at odds with our data that phagocytosis is impaired as a consequence of silencing ARHGAP25, as was the case also for SH3BP1 and ARHGAP12. However, the role of ARHGAP25 in the ingestion of serum-opsonized particles is in all likelihood very different to its role in FcγR-mediated phagocytosis; the complement receptor activated during phagocytosis of serum-coated targets operates via RhoA^30^,^32^, in contrast to the Fcγ receptors engaged in our studies, which function primarily via Rac1/2 and Cdc42 (refs 8, 9, 13). Indeed, no increase in phagocytic efficiency was observed in ARHGAP25-silenced cells when the yeast particles were opsonized with heat-inactivated serum^23^, wherein complement is inhibited.

Why is phagocytosis of small targets possible in the absence of PI3K activity and how do such phagosomes proceed to fuse with endomembranes? As discussed earlier, we believe that sealing of small phagosomes can be completed with little need for membrane expansion and without incurring exhaustion of components of the actin cytoskeletal machinery. Thus, the sealing of small phagosomes would be expected to be less reliant on PI3K-driven actin disassembly, compared with that of large ones. Indeed, as documented in Fig. 6, small phagocytic targets trigger little PtdIns(3,4,5)P_3_ biosynthesis on engagement of Fcγ receptors. As a consequence, the RhoGAPs that are normally responsive to PI3K stimulation do not translocate to small cups, and phagosomes seal while still maintaining a ring of periphagosomal actin. Once phagosomes become internalized, detachment of the cortical skeleton likely occurs as a result of the hydrolysis of PtdIns(4,5)P_2_ (ref. 33). This inositide not only anchors a variety of adaptors that link F-actin filaments to the membrane^34^,^35^, but also electrostatically targets and retains Rho GTPases at the plasmalemma^36^,^37^. Thus, fission of small phagosomes is followed by PtdIns(4,5)P_2_ catabolism^31^, and consequently actin disassembly. Likely candidates for the late stage of PtdIns(4,5)P_2_ breakdown in small phagosomes include the 5′-phosphatases OCRL and Inpp5b, which translocate to sites of phagocytosis^38^,^39^,^40^.

## Methods

### Reagents

Polystyrene microspheres (8.31 or 1.58 μm in diameter) functionalized with divinylbenzene were obtained from Bangs Laboratories, Inc. Sheep erythrocytes (10% suspension) were purchased from MP Biomedicals. LY294002, rapamycin, DMSO and human-serum IgG were from Sigma-Aldrich. Fluorescent antibodies against human and rabbit IgG, including Cy5-, Cy3- and DyLight 488-conjugates were from Jackson ImmunoResearch Labs. Anti-sheep red blood cell antibodies were purchased from Cedarlane Laboratories. Paraformaldehyde (16% w/v) was from Electron Microscopy Sciences. Fluorescent phalloidin was from Molecular Probes (Life Technologies). Rabbit polyclonal antibodies against mouse ARHGAP12, ARHGAP25 and SH3BP1 were obtained from Sigma-Aldrich (product number HPA000412), Thermo Scientific (catalogue number PA5-24791) and Proteintech (catalogue number 20541-1-AP), respectively. For the dilutions at which the antibodies were used, see the Immunoblotting section below.

### Plasmids

cDNAs encoding RhoGAP proteins were selected by the presence of a RhoGAP Pfam domain (PF00620) within putative coding regions from the Ensembl database. When multiple isoforms existed for a particular RhoGAP, the isoform encompassing all known domains carried by the protein was considered the representative longest isoform, and therefore chosen as a target for cloning. The cDNAs were sourced from the Mammalian Gene Collection (MGC) or synthesized (GenScript) when a suitable full-length isoform did not exist. The cDNAs were cloned in-frame into AscI/PacI sites within Creator donor plasmids, and recombined into Creator expression vectors to generate N-terminal mCitrine-tagged fusion proteins on expression. Where PDZ-binding motifs were predicted (as in BCR and ABR), the cDNAs were cloned with a stop codon immediately after the coding sequence.

Active (GTP-bound) Rac and Cdc42 were detected with PAK(PBD)–YFP^16^, a plasmid encoding the PBD of PAK fused to YFP. Polymerized actin was visualized using Lifeact–mRFP, as described earlier^12^. Rac1(Q61L)–HR_tail_, encoding constitutively active Rac1 conjugated to GFP and modified to carry the hydrophobic tail of H-Ras in lieu of its polybasic domain, was used to induce sustained Rac1 signalling at the plasmalemma^36^. Likewise, Cdc42(G12V) was used to induce constitutive signalling by Cdc42. This protein was encoded in the YFP–Cdc42(V12) plasmid, a gift from Dr Joel Swanson (Addgene plasmid #11399). A construct encoding PM–RFP^41^ was used to label the plasma membrane. The membrane-targeting component of the rapamycin-inducible heterodimerization system, consisting of the N-terminal 11 amino acids of Lyn kinase conjugated to a complementary rapamycin-biding domain (Lyn_11_–FRB) was a generous gift of Dr G. Di Paolo (Columbia University)^42^. Rapamycin-recruitable Tiam1 (YFP–FKBP–Tiam1) was obtained from Addgene (plasmid #20154)^18^. The distribution of PtdIns(3,4,5)P_3_ was monitored using the PH domain of Akt^43^.

### Isolation of primary macrophages and cell culture

Peripheral blood mononuclear cells were obtained from healthy volunteers and isolated by density gradient centrifugation using Lympholyte-H (Cedarlane). Monocytes were purified by adherence to 1.8 cm glass coverslips in 12-well plates (3 × 10^6^ peripheral blood mononuclear cells per coverslip). Cells were cultured for 5 days at 37 °C under 5% CO_2_ in Roswell Park Memorial Institute (RPMI) 1640 medium supplemented with 10% heat-inactivated fetal bovine serum (Wisent Inc.), antibiotics (Multicell) and 25 ng ml^−1^ macrophage colony-stimulating factor. Following this differentiation protocol, macrophages adhered firmly to the coverslips and non-adherent cells were removed by washing.

The murine macrophage line RAW 264.7 was obtained from the American Type Culture Collection (ATCC) and grown in RPMI 1640 supplemented with 5% heat-inactivated fetal bovine serum at 37 °C under 5% CO_2_.

### DNA transfection

For transient transfections, nearly confluent monolayers of RAW 264.7 cells were lifted by careful scraping and plated onto 1.8 cm glass coverslips at a density of 1 × 10^5^ cells per coverslip. Macrophages were allowed to recover for 18–24 h and then transfected with FuGENE HD (Promega) according to the manufacturer's instructions. Briefly, 2 μg of plasmid DNA and 6 μl of the transfection reagent were mixed in 100 μl of serum-free RPMI and allowed to sit for 15 min. The mix was then added to 500 μl of RPMI and distributed equally into four wells of a 12-well plate. Cells were typically used for experimentation 18 h after transfection.

Alternatively, high levels of protein expression were achieved by electroporating plasmid DNA with the Neon Transfection System (Life Technologies) according to the manufacturer's protocol. Briefly, adherent RAW 264.7 cells or primary macrophages were lifted by gentle scraping or by a 10-min exposure to Accutase (Innovative Cell Technologies), respectively. Cells were then counted and sedimented at 300 *g* for 5 min; 5 × 10^5^ cells were resuspended in 100 μl of buffer R and incubated with 10 μg plasmid DNA; and 100 μl of the cell mix was then subjected to electroporation using a single 20-millisecond pulse of 1750 V (RAW 264.7 cells) or two sequential 30-millisecond pulses of 1,100 V (primary macrophages). Electroporated macrophages were immediately transferred to RPMI for seeding into coverslips.

### Gene silencing

siRNA directed against *SH3BP1*, *ARHGAP12* or *ARHGAP25* were purchased from Dharmacon. For each transcript targeted, oligonucleotides were obtained as a mixture of four different siRNAs (SMARTpools).

The sequences targeted in *SH3BP1* were:

1. 5′- ccucugaccucuacgauga -3′
2. 5′- ucacugaccucacucgaca -3′
3. 5′- ucgaggcgcugauacagaa -3′
4. 5′- gcagaggagcaggauguaa -3′

The sequences targeted in *ARHGAP12* were:

1. 5′- ggaguaugauuaugaguau -3′
2. 5′- guuuagauguugaugguau -3′
3. 5′- gcugaaaacucgacaagga -3′
4. 5′- gcaggacaagcguauauug -3′

The sequences targeted in *ARHGAP25* were:

1. 5′- caagaacucuggcgaggau -3′
2. 5′- ggaaaucagccuucgaaau -3′
3. 5′- gaacuaugucccaagacuu -3′
4. 5′- uaaaaggacucaaacgcuu -3′

Oligonucleotides were delivered by electroporating 5 × 10^5^ RAW 264.7 cells with 200 pmol of the siRNA pool with the Neon system, using a single 20-millisecond pulse of 1750 V. Electroporated cells were allowed to recover for 48 h before being lifted once again for a second round of electroporation. Knockdown efficiency and phagocytosis were assessed 96 h after the initial electroporation.

### Immunoblotting

RAW 264.7 cells transfected with siRNA directed against one of the three RhoGAP candidates or with a non-targeting siRNA control were washed with PBS and lysed with cold RIPA buffer (Sigma-Aldrich) supplemented with protease- and phosphatase-inhibitor tablets (Roche). 40 μg of total protein were loaded per lane of a 12% SDS–polyacrylamide gel electrophoresis and separated by electrophoresis, before being transferred to methanol-activated polyvinylidene fluoride membranes. Membranes were blocked in Tris-buffered saline (TBS) supplemented with 0.1% Tween 20 and 5% bovine serum albumin for 30 min. This solution was also used to dilute the primary anti-RhoGAP and anti-rabbit horseradish peroxidase (HRP)-conjugated secondary antibodies, which were incubated for 2 h and 45 min, respectively. Primary antibodies against ARHGAP12, ARHGAP25 and SH3BP1 were used at 1:250, 1:1,000 and 1:1,000 dilutions, respectively. HRP-conjugated antibodies were used at a 1:10,000 dilution. Membranes exposed to ECL western blotting substrate (GE Life Sciences) were visualized using the Odyssey Fc (LI-COR) system. Brightness/contrast parameters were adjusted globally across the entire image using the LI-COR Image Studio software. Immunoblots were cropped for presentation; see Supplementary Fig. 6 for uncropped images.

### Phagocytosis

For all FcγR-mediated phagocytosis assays, ≈1 × 10^5^ RAW 264.7 cells were seeded onto 1.8 cm glass coverslips and allowed to grow for 24 h. Divinylbenzene-coated polystyrene beads were diluted 10-fold in PBS and opsonized by incubating with human IgG (final IgG concentration=5 mg ml^−1^) for 60 min at room temperature. Excess IgG was removed by washing the beads twice with 1 ml of PBS. Alternatively, sheep erythrocytes were opsonized by incubating 200 μl of a 10% suspension with 5 μl of a rabbit anti-sheep red blood cell antibody for 60 min at room temperature. Excess IgG was removed by washing the erythrocytes 3x with PBS. Beads and sheep erythrocytes were then labelled with fluorescent antibodies against human or rabbit IgG, respectively, and resuspended in 200 μl PBS. Phagocytosis was initiated by adding 15 μl or 5 μl of the bead or erythrocyte suspension, respectively, to each well of a 12-well plate and sedimenting the targets onto the macrophage monolayer by centrifugation (300 *g*, 30 s). Where indicated, cells were treated with either DMSO or LY294002 (100 μM) for 10 min before initiating phagocytosis. Cells were allowed to phagocytose for the indicated times before fixing with paraformaldehyde (PFA; 4%). Particles that failed to be internalized were visualized by staining with secondary antibodies against IgG conjugated to a fluorophore different from the one originally used for labelling the phagocytic targets. To stain actin filaments, cells were permeabilized with Triton X-100 (0.1%) for 5 min and incubated with fluorescent phalloidin (Molecular Probes, 1:1,000 dilution) for 45 min.

### Microscopy and image analysis

All fluorescence imaging was performed with spinning-disk confocal microscopes (Quorum Technologies). The systems in use in our laboratory are based on an Axiovert 200M microscope (Carl Zeiss) equipped with a × 63 oil-immersion objective (NA 1.4) and a × 1.5 magnifying lens. These units carry a motorized XY stage (Applied Scientific Instrumentation), a Piezo Z-focus drive and diode-pumped solid-state lasers emitting at 440, 491, 561, 638 and 655 nm (Spectral Applied Research). Images were recorded with back-thinned, cooled charge-coupled device cameras (Hamamatsu Photonics) under control of the Volocity software (version 6.2.1; PerkinElmer). Fluorescence intensity measurements and correction of brightness/contrast were performed with ImageJ (version 1.48; National Institutes of Health). Brightness and contrast parameters were adjusted across the entire image and without altering the linearity of mapped pixel values.

### Reverse transcription polymerase chain reaction

One-step RT–PCR (Invitrogen) was used to ascertain whether individual RhoGAPs were transcribed endogenously. For each RT–PCR reaction, RNA was purified from ∼8 × 10^5^ RAW 264.7 cells using the RNeasy Mini kit (Qiagen) according to the manufacturer's instructions. Reverse transcription of RhoGAP messages or of GAPDH (positive control) was performed using sequence-specific primers that spanned exon–exon junctions and 1 μg of purified RNA template. The reverse transcription reaction was carried out at 55 °C and allowed to proceed for 30 min, before increasing the temperature to 94 °C to inactivate reverse transcriptase and initiate exponential amplification by Taq polymerase. PCR amplification continued for 19 cycles at denaturing, annealing and extension temperatures of 94 °C, 50 °C and 68 °C, respectively. Finally, amplicons were visualized by electrophoresis using agarose gels prestained with ethidium bromide. An uncropped image of the agarose gel is presented in Supplementary Fig. 6a. The primer sequences used for cDNA synthesis in this study are provided in Supplementary Table 1.

### Rapamycin heterodimerization system and Tiam1 translocation

Rapamycin-inducible recruitment of the GEF domain of Tiam1 to the plasma membrane was performed as described earlier^18^,^44^,^45^. In brief, the heterodimerization system consists of two separate constructs: one encodes a polypeptide with the membrane-targeting sequence of Lyn, which associates constitutively with the plasmalemma, the other encodes the GEF domain of Tiam1 conjugated to YFP, and is normally cytosolic. Both proteins carry complementary rapamycin-binding domains, and addition of rapamycin induces the interaction between them, thereby supporting the rapid and virtually irreversible translocation of recombinant Tiam1 to the plasmalemma.

### Statistical analysis

For analysis of significance, at least three independent experiments of each type were performed, counting multiple cells in each experiment, as indicated in the text or figures. A sample size of at least 20 cells was chosen so that ∼100 phagosomes/nascent phagocytic cups could be quantified per condition. Values are reported as means of the experiments±s.e.m. Statistical parameters were assessed with GraphPad Prism 5c software (GraphPad Software, Inc.). The significance of differences was determined performing unpaired *t*-tests, using the routines built in GraphPad Prism.

## Additional information

**How to cite this article:** Schlam, D. *et al*. Phosphoinositide 3-kinase enables phagocytosis of large particles by terminating actin assembly through Rac/Cdc42 GTPase-activating proteins. *Nat. Commun.* 6:8623 doi: 10.1038/ncomms9623 (2015).

## Supplementary Material

Supplementary Figures and Supplementary Table Supplementary Figures 1-6 and Supplementary Table 1

Supplementary Movie 1 Actin cytoskeletal dynamics during phagocytosis of large phagocytic targets. RAW 264.7 macrophages were transfected with Lifeact-mRFP to visualize actin polymerisation. Transfectants were challenged with 8.3-μm IgG-opsonized beads (indicated with a star) immediately before being imaged live at 37°C by spinning-disk confocal microscopy. Frames were acquired every 10.3 sec (real time is indicated by the clock on the lower right). Disassembly of actin networks at the base of the phagocytic cup is signaled with a red arrow.

Supplementary Movie 2 PI3K activity is necessary for the clearance of F-actin from the base of the phagocytic cup during internalisation of large phagocytic targets. RAW 264.7 macrophages were transfected with Lifeact-mRFP to visualize sites of actin polymerisation. Transfectants were treated with the PI3K inhibitor LY294002 for 10 min immediately prior to being challenged with 8.3-μm IgG-opsonized beads (indicated with a star). Phagocytosis was imaged live at 37°C by spinning-disk confocal microscopy. Frames were acquired every 13.5 sec (real time is indicated by the clock on the lower right).

Supplementary Movie 3 Forced recruitment of the GEF domain of Tiam1 to the plasma membrane is not sufficient to preclude actin clearance. RAW 264.7 macrophages were transiently co-transfected with vectors encoding Lifeact-mRFP (left panel) and a recruitable form of the Rac1 GEF domain of Tiam1 (right panel). Addition of rapamycin triggers translocation of recombinant Tiam1 to the plasmalemma, where its rapamycin-binding domain (FKBP) interacts with a second, complementary rapamycin-binding moiety (colourless in this case). Immediately after inducing Tiam1 translocation to the plasma membrane, macrophages were challenged with 8.3-μm IgG-coated microspheres and imaged live at 37°C by spinning-disk confocal microscopy. White arrows point to phagocytic targets that are engulfed despite sustained association of Tiam1 with phagocytic membranes. Note that actin disassembles from the surface of internalised phagosomes following fission, whereas Tiam1 remains associated with these compartments.

## Figures and Tables

**Table 1:** Collated data of the screening of RhoGAPs involved in actin remodelling.

**Gene name**	**Accumulation at phagocytic cups**	**Endogenously expressed**	**PI3K-dependent recruitment**
*ARHGAP9*	Yes	No	No
*ARHGAP12*	Yes	Yes	Yes
*ARHGAP24*	Yes	No	No
*ARHGAP25*	Yes	Yes	Yes
*BCR*	Yes	No	Yes
*GRLF1*	Yes	Yes	No
*MYO9B*	Yes	Yes	No
*OPHN1*	Yes	No	No
*PIK3R1*	Yes	Yes	No
*SH3BP1*	Yes	Yes	Yes

The table indicates which RhoGAPs translocate to sites of particle engagement in response to IgG-coated targets, whether they are endogenously expressed in RAW 264.7 macrophages, and the dependency of their recruitment to phagocytic cups on PI3K signals.
