# Supplementary material for: Phosphoinositide 3-kinase enables phagocytosis of large particles by terminating actin assembly through Rac/Cdc42 GTPase-activating proteins
Source: Nat Commun. 2015 Oct 14;6:8623. doi: 10.1038/ncomms9623 (PMC4634337; doi:10.1038/ncomms9623)
Supplement: Supplementary Figures and Supplementary Table — Supplementary Figures 1-6 and Supplementary Table 1 [file ncomms9623-s1.pdf]

SUPPLEMENTARY INFORMATION

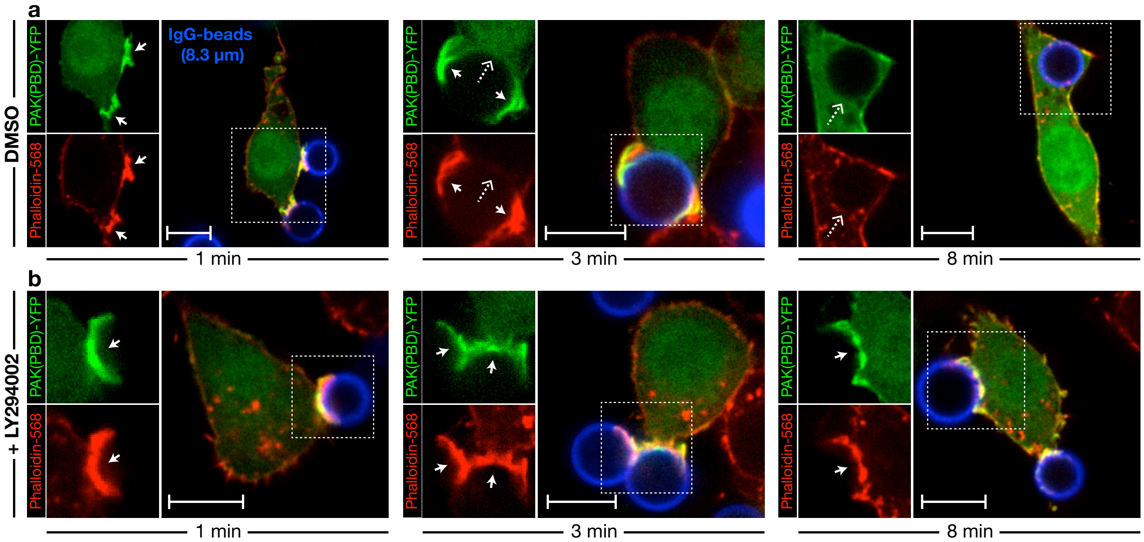

Supplementary Figure 1: Confocal micrographs of RAW 264.7 macrophages transfected with PAK(PBD)-YFP (shown in green), a biosensor for active Rac/Cdc42, during phagocytosis of 8.3-µm IgG-beads. Prior to phagocytosis, cells were treated with DMSO (**a**) or LY294002 (**b**) for 10 min. Phagocytosis was allowed to proceed for the indicated times, before fixing and staining F-actin with phalloidin (shown in red). All phagocytic targets were stained with a Cy5-conjugated secondary antibody (shown in blue) prior to phagocytosis. Insets (boxed regions) show magnified views of the phagocytic cup. Scale bar, 10 µm. Micrographs are representative of 3 independent experiments; at least 20 cells were assessed per replicate.

Name & GeneID (human, mouse, rat, chimpanzee)	mCitrine-RhoGAP	PM-RFP	RhoGAP + PM-RFP + IgG-E	Subcellular localisation at rest	Distribution during phagocytosis	Phagocytic cup recruitment (score)
Inositol polyphosphate-5-phosphatase, 75kDa (INPP5B) 3633	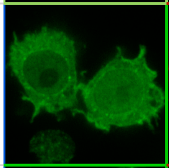	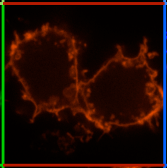	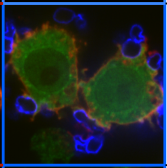	Ubiquitous distribution; subtle accumulation at nuclear and plasma membranes	Present at cup but already found at plasma membrane prior to particle engagement	None-to -subtle: 1
Oculocerebrorenal syndrome of Lowe (OCRL) 4952	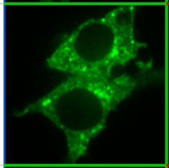	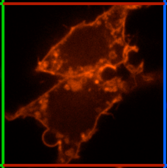	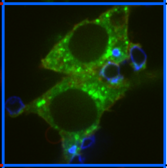	Punctated and primarily found in perinuclear compartments. Also present at plasma membrane	Present at cup but already found at plasma membrane prior to particle engagement	None-to -subtle: 1
Rho GTPase activating protein 36 (ARHGAP36) 158763	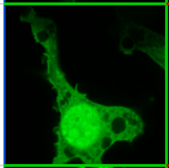	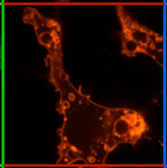	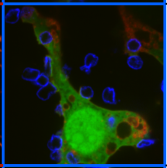	Primarily nuclear. Also cytosolic and plasma membrane bound	Present at cup but already found at plasma membrane prior to particle engagement	None-to -subtle: 1
Rho GTPase activating protein 36 (ARHGAP36) short 158763	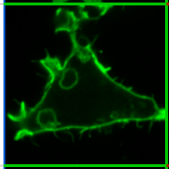	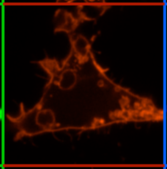	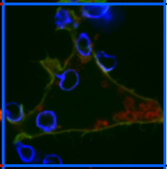	Exclusively plasma membrane	Present at cup but already found at plasma membrane prior particle engagement. Increase in GAP fluorescence	Subtle: 2
Rho GTPase activating protein 40 (ARHGAP40) 343578	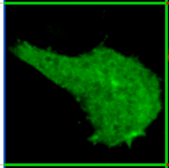	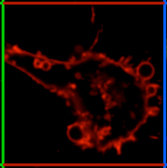	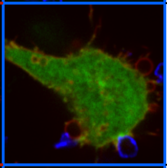	Ubiquitous distribution	Present at cup but already found at plasma membrane prior to particle engagement	None-to -subtle: 1
Rho GTPase activating protein 19 (ARHGAP19) 84986	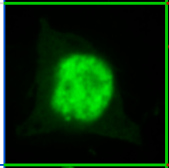	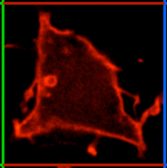	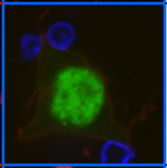	Exclusively nuclear and and somewhat plasma membrane	Subcellular localisation is a compartment distant from nascent cups	None: 0
Deleted in liver cancer 1 (DLC1) 10395	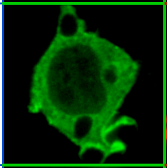	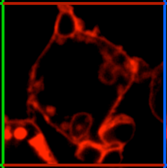	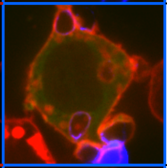	Mainly cytosolic but also found at plasma membrane	Present at cup but already found at plasma membrane prior to particle engagement.	None-to -subtle: 1
StAR-related lipid transfer (START) domain containing 8 (STARD8) 9754	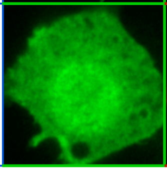	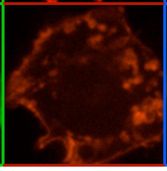	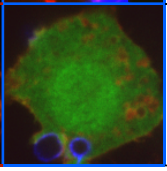	Ubiquitous distribution	Present at cup but already found at plasma membrane prior to particle engagement	None-to -subtle: 1

StAR-related lipid transfer (START) domain containing 13 (STARD13) 90627	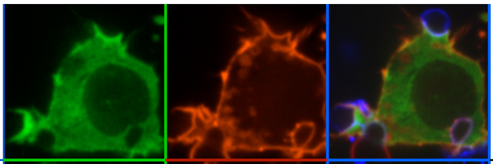	Cytosolic and plasma membrane	Present at cup but already found at plasma membrane prior to particle engagement	Subtle-to-moderate: 3
Rho GTPase activating protein 21 (ARHGAP21) 57584	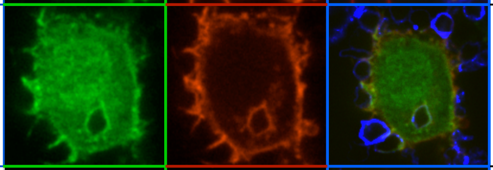	Ubiquitous distribution but primarily plasma membrane and nucleus	Marked retention at sealed phagosome and mild accumulation at plasma membrane	Subtle-to-moderate: 3
Myosin IXB (MYO9B) 4650	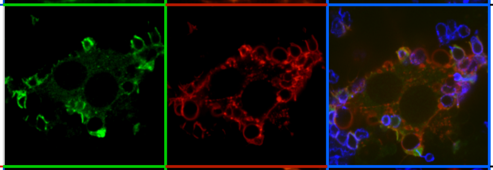	Phagocytic cups and sealed phagosomes nearly exclusively	Pronounced accumulation at phagocytic cups and internalized phagosomes	Moderate-to-exceptional 5
GEM interacting protein (GMIP) 51291	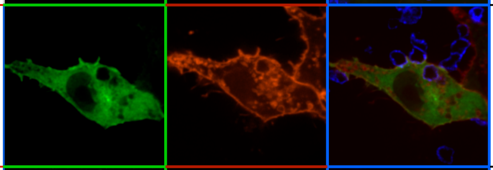	Cytosolic and plasma membrane	Present at cup but already found at plasma membrane prior to particle engagement	Subtle: 2
Rho GTPase activating protein 29 (ARHGAP29) 9411	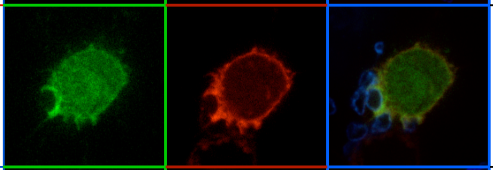	Cytosolic and plasma membrane	Present at cup but already found at plasma membrane prior to particle engagement	Subtle: 2
Rac GTPase activating protein 1 (RACGAP1) 29127	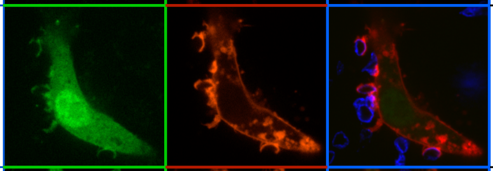	Cytosolic and nuclear	Present at cup but already found at plasma membrane prior to particle engagement	None-to -subtle 1
Histocompatibility (minor) HA-1 (HMHA1) 23526	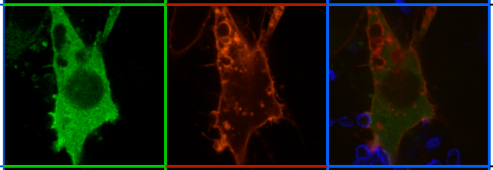	Cytosolic and plasma membrane	Present at cup but already found at plasma membrane prior to particle engagement	Subtle: 2
Synapse defective 1, Rho GTPase, homolog 2 (*C. elegans*) (SYDE2) 84144	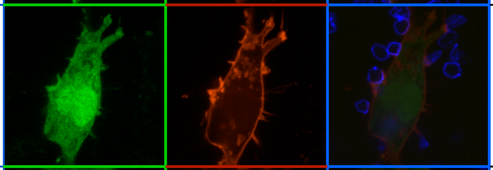	Primarily nuclear. Also cytosolic and plasma membrane bound	Present at cup but already found at plasma membrane prior to particle engagement	Subtle: 2
RalA binding protein 1 (RALBP1) 10928	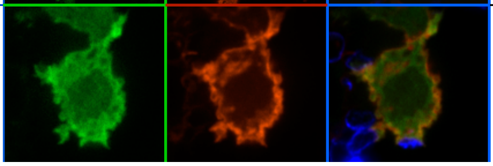	Ubiquitous distribution	Present at cup but already found at plasma membrane prior to particle engagement	None-to -subtle 1

Family with sequence similarity 13, member A (FAM13A) 10144	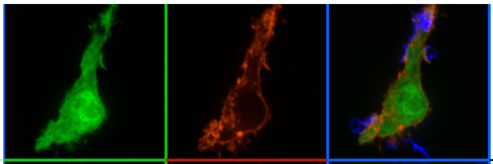	Primarily nuclear but ubiquitously present	Present at cup but already found at plasma membrane prior to particle engagement	Subtle: 2
Family with sequence similarity 13, member B (FAM13B) 51306	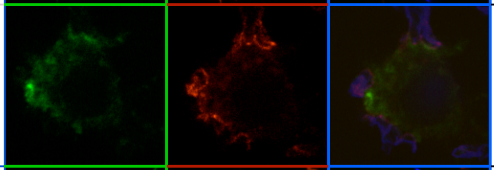	Cytosolic and plasma membrane	Cytosolic and plasma membrane	Subtle: 2
Chimerin 2 (CHN2) 1124	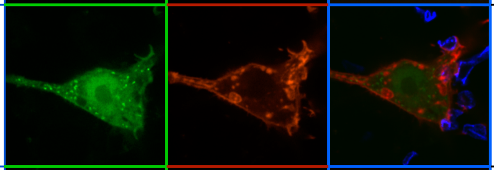	Ubiquitous distribution + punctae	Present at cup but already found at plasma membrane prior to particle engagement	Subtle: 2
Chimerin 1 (CHN1) 1123	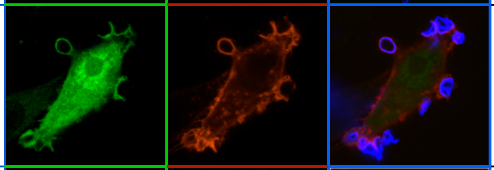	Primarily nuclear. Also cytosolic and plasma membrane bound	Present at cup but already found at plasma membrane prior to particle engagement	Subtle-to-moderate: 3
ArfGAP with RhoGAP domain, ankyrin repeat and PH domain 1 (ARAP1) 116985	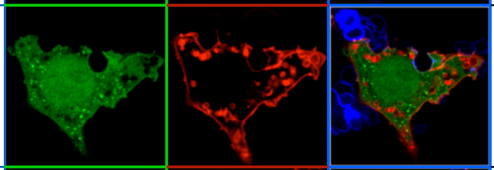	Ubiquitous distribution + punctae	Present at cup but also ubiquitously distributed	None-to -subtle 1
ArfGAP with RhoGAP domain, ankyrin repeat and PH domain 3 (ARAP3) 116984	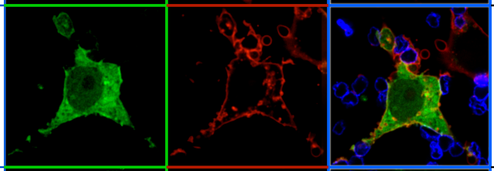	Plasma membrane and cytosolic	Subtle increase at phagocytic cup but already found at plasma membrane prior to particle engagement	Subtle: 2
Rho GTPase-activating protein 20 (ARHGAP20) 57569	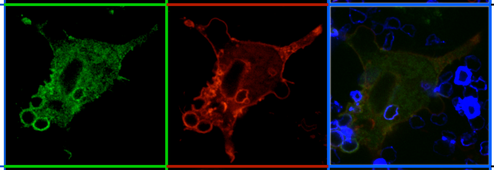	Plasma membrane and cytosolic	Moderate increase at phagocytic cup but already found at plasma membrane prior to particle engagement	Subtle-to-moderate: 3
Rho GTPase activating protein 23 (ARHGAP23) 57636	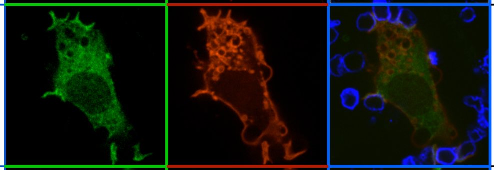	Primarily plasma membrane but also cytosolic to a lesser extent	Present at cup but already found at plasma membrane prior to particle engagement	Subtle-to-moderate: 3
Rho GTPase activating protein 15 (ARHGAP15) 55843	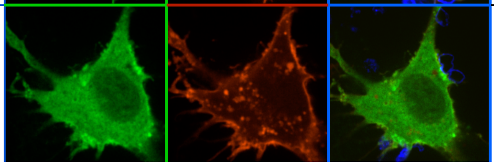	Plasma membrane and cytosol	Present at cup but already found at plasma membrane prior to particle engagement	Subtle-to-moderate: 3

Rho GTPase activating protein 24 (ARHGAP24) 83478	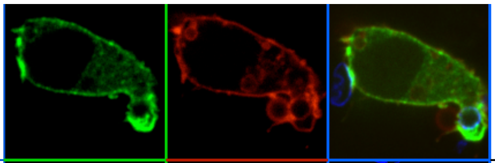	Primarily plasma membrane	Marked accumulation at base of phagocytic cup although already at plasma membrane	**Moderate 4**
Oligophrenin 1 (OPHN1) 4983	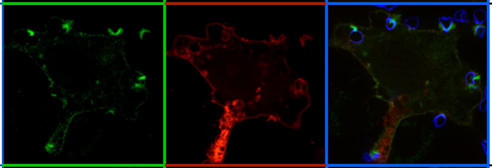	Primarily plasma membrane	Recruitment to phagocytic cup exclusively	**Exceptional 6**
Rho GTPase activating protein 25 (ARHGAP25) 9938	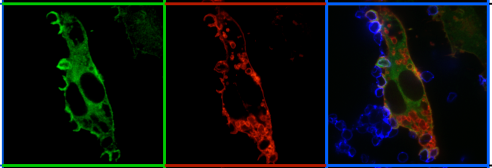	Plasma membrane and cytosol	Moderate accumulation at nascent cups. Increase is less obvious at the base of the cup than at the top	**Moderate 4**
Rho GTPase activating protein 22 (ARHGAP22) 58504	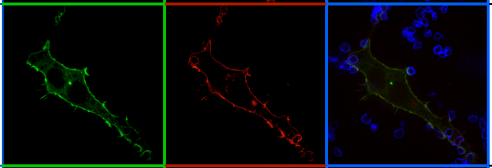	Exclusively at the plasma membrane	No accumulation; already present at the plasma membrane	**None-to -subtle 1**
GEF/GAP breakpoint cluster region (BCR) 613	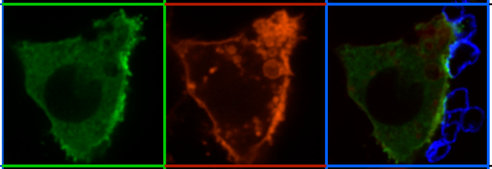	Plasma membrane and cytosol	Robust recruitment to base of the cup	**Moderate 4**
GEF/GAP active BCR-related gene (ABR) 29	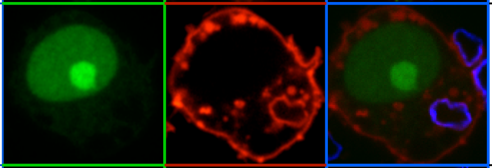	Exclusively nuclear	Subcellular localisation is a compartment distant from nascent cups	**None: 0**
Rho GTPase activating protein 27 (ARHGAP27) 201176	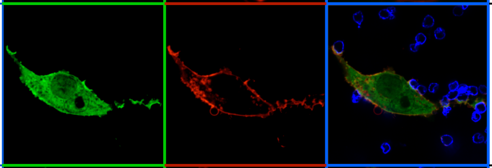	Plasma membrane and cytosol	Present at cup but already found at plasma membrane prior to particle engagement	**Subtle-to-moderate: 3**
Rho GTPase activating protein 12 (ARHGAP12) 94134	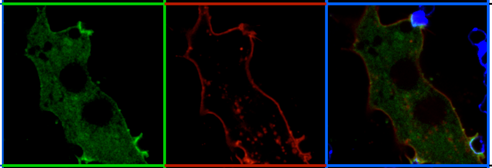	Plasma membrane and cytosolic	Present at cup but already found at plasma membrane prior to particle engagement	**Moderate 4**
Rho GTPase activating protein 8 (ARHGAP8) 23779	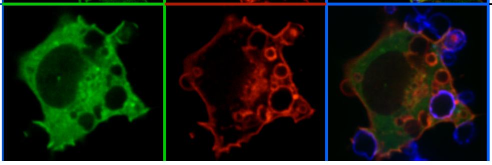	Cytosolic	No obvious recruitment; ubiquitously present throughout the cell	**Subtle: 2**

Rho GTPase activating protein 1 (ARHGAP1) 392	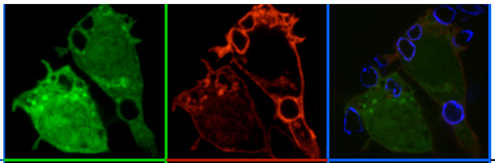		Ubiquitous localisation and formation of punctae.	No obvious recruitment; ubiquitously present throughout the cell	Subtle: 2
Rho GTPase activating protein 44 (ARHGAP44) 9912	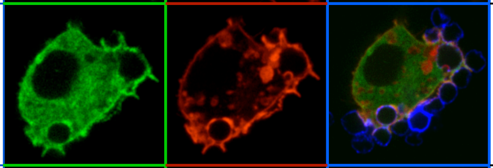		Cytosolic and plasma membrane	Slight increase at the base of the phagocytic cup, although constitutively present at the plasma membrane	Subtle: 2
Rho GTPase activating protein 17 (ARHGAP17) 55114	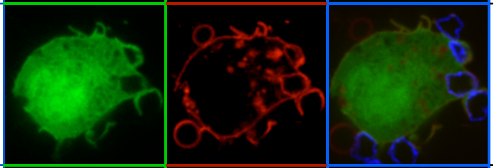		Primarily nuclear. Also cytosolic and plasma membrane bound	No obvious recruitment; ubiquitously present throughout the cell	Subtle: 2
SH3-domain binding protein 1 (SH3BP1) 23616	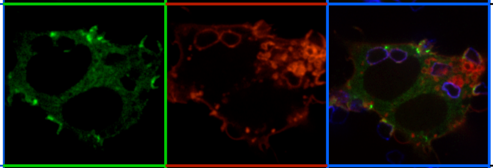		Diffuse signal throughout cytosol	Marked recruitment to nascent phagocytic cups	Moderate-to-exceptional 5
SLIT-ROBO Rho GTPase activating protein 1 (SRGAP1) 57522	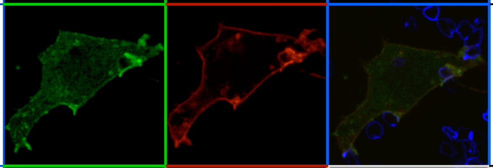		Plasma membrane	Possible recruitment to nascent cups	Subtle: 2
Rho GTPase activating protein 4, isoform 2 (ARHGAP4) 393	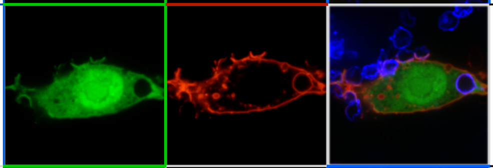		Primarily nuclear but also cytosolic and plasma membrane bound	No recruitment to phagocytic cups; ubiquitously present throughout the cell	Subtle: 2
SLIT-ROBO Rho GTPase activating protein 3, isoform a (SRGAP3) 9901	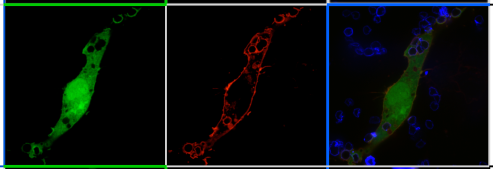		Primarily nuclear but also cytosolic and plasma membrane bound	No recruitment to phagocytic cups; ubiquitously present throughout the cell	Subtle: 2
SLIT-ROBO Rho GTPase activating protein 2, isoform c (SRGAP2) 23380	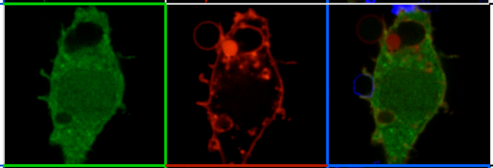		Ubiquitous distribution	No recruitment; already present at plasma membrane	None-to -subtle 1
Rho GTPase activating protein 26 (ARHGAP26) 23092	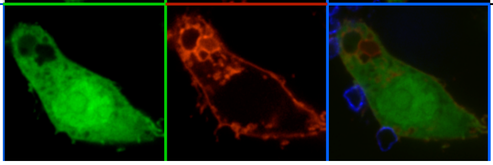		Primarily plasma membrane and nuclear but to a lesser extent cytosolic	No recruitment; already present at plasma membrane	Subtle: 2

Rho GTPase activating protein 9 (ARHGAP9) 64333	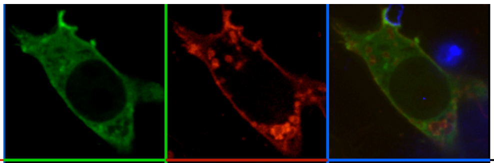	Primarily plasma membrane and nuclear but to a lesser extent cytosolic	Recruitment is more obvious in some cells, although already present at the PM prior to particle engagement	**Moderate 4**
Phosphoinositide-3-kinase, regulatory subunit 1 (alpha) (PIK3R1) 5295	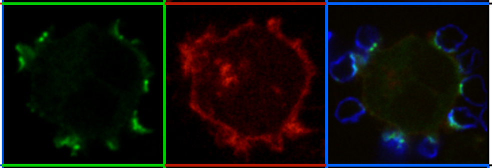	Diffuse signal throughout cytosol	Signal exclusively at the base of phagocytic cup	**Exceptional 6**
Phosphoinositide-3-kinase, regulatory subunit 2 (beta) (PIK3R2) 5296	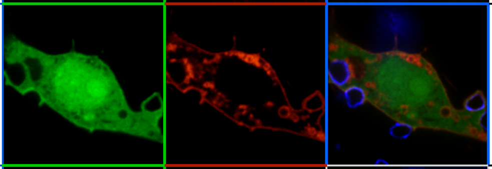	Ubiquitous distribution; subtle accumulation at nuclear and plasma membranes	Subtle accumulation at the base of the cup	**Subtle: 2**
DEP domain containing 1 (DEPDC1) 55635	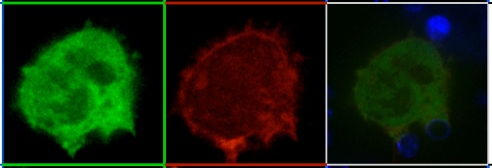	Low expression levels; ubiquitously distributed	No recruitment; already present at plasma membrane	**None-to -subtle 1**
DEP domain containing 1B (DEPDC1B) 55789	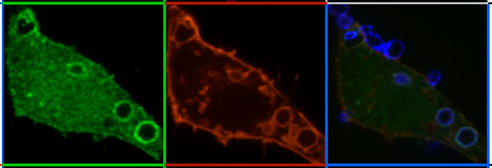	Low expression levels; ubiquitously distributed	Marked retention at sealed phagosome and mild accumulation at plasma membrane	**Subtle: 2**
Rho GTPase activating protein 39 (ARHGAP39) 80728	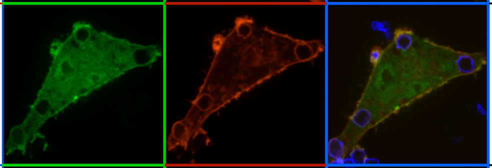	Primarily nuclear. but also cytosolic	Moderate recruitment to the cup proportionally to plasma membrane increase	**Subtle-to-moderate: 3**
Rho GTPase activating protein 35 (ARHGAP35; GRLF1) 2909	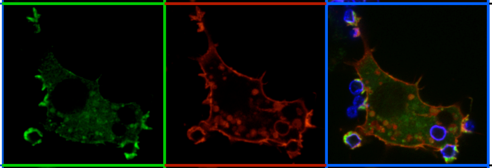	Diffuse signal throughout cytosol	Signal exclusively at the base of phagocytic cup	**Exceptional 6**
Rho GTPase activating protein 5 (ARHGAP5) 394	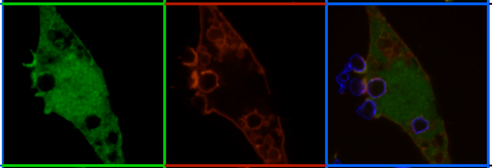	Diffuse signal throughout cytosol	Slight recruitment to the cup proportionally to plasma membrane increase	**None-to -subtle 1**
T-cell activation RhoGTPase activating protein (TAGAP) 117289	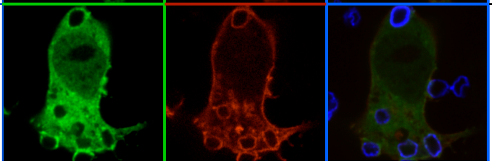	Ubiquitous distribution	Slight recruitment to the cup proportionally to plasma membrane increase	**Subtle: 2**

Rho GTPase activating protein 32 (ARHGAP32) 9743	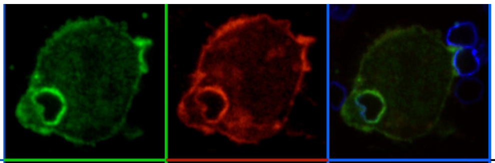	Ubiquitous distribution but primarily plasma membrane	Present at cup but already found at plasma membrane; obvious retention at phagosomal membrane	**Subtle-to-moderate: 3**
Rho GTPase activating protein 6 (ARHGAP6) 395	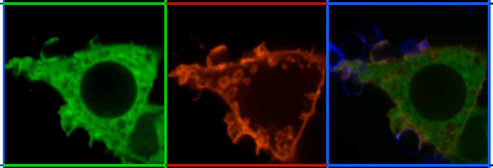	Cytosolic and plasma membrane	No recruitment; already present at plasma membrane	**Subtle: 2**
Synapse defective 1, Rho GTPase, homolog 1 (*C. elegans*), isoform b (SYDE1) 85360	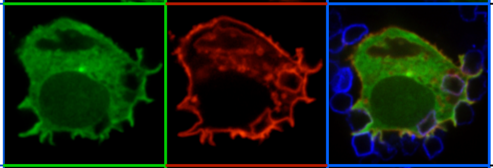	Cytosolic and plasma membrane	No recruitment; already present at plasma membrane	**Subtle: 2**
Synapse defective 1, Rho GTPase, homolog 1 (*C. elegans*), isoform a (SYDE1) 85360	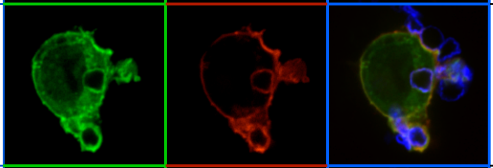	Plasma membrane and cytosol	Present at cup but already found at plasma membrane prior particle engagement	**Subtle-to-moderate: 3**
Rho GTPase activating protein 30 (ARHGAP30) 257106	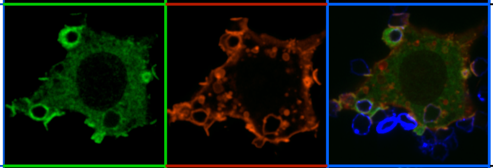	Plasma membrane and cytosol	Low signal intensity in the cytosol but strong recruitment to cups + retention at sealed phagosome	**Subtle-to-moderate: 3**
Rho GTPase activating protein 31 (ARHGAP31) 57514	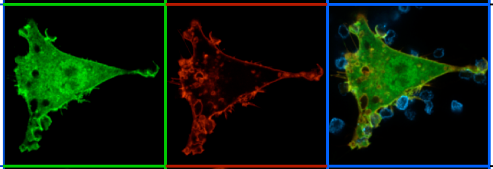	Plasma membrane, nucleus and cytosol	Present at cup but already found at plasma membrane prior particle engagement. Mild retention at seal phagosome	**Subtle-to-moderate: 3**
Rho GTPase activating protein 42 (ARHGAP42) , Cdc42 GTPase-activating protein 143872	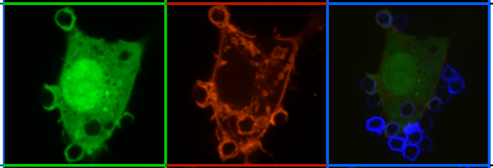	Plasma membrane, nucleus and cytosol	Present at cup but already found at plasma membrane prior particle engagement.	**Subtle-to-moderate: 3**
Rho GTPase activating protein 28 (ARHGAP28) 79822	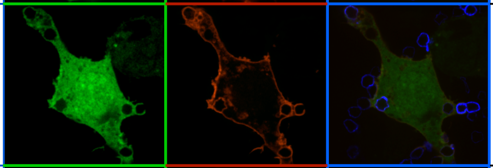	Ubiquitous distribution	Present at cup but already found at plasma membrane prior particle engagement.	**Subtle: 2**
Rho GTPase activating protein 18 (ARHGAP18) 93663	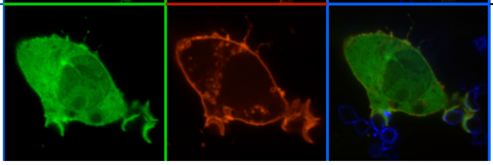	Ubiquitous distribution	Present at cup but already found at plasma membrane prior particle engagement.	**Subtle: 2**

Supplementary Figure 2: Subcellular localization of RhoGAP-family members at rest and during phagocytosis. RhoGAP names and their corresponding gene IDs (National Centre for Biotechnology Information) are indicated in the first column. Names have been colour-coded to indicate the species from which the primary sequence was derived. The distribution of mCitrine-tagged RhoGAPs was followed by confocal microscopy in RAW 264.7 macrophages challenged with IgG-opsonized erythrocytes (IgG-E, shown in blue in column 4). In order to distinguish *bona fide* accumulation of the RhoGAP in question from bulk enrichment of plasma membrane at sites of phagocytosis, cells were transfected with the plasmalemmal marker PM-RFP. The relative extent of RhoGAP accumulation at phagocytic cups was assessed by ratiometric analysis relative to PM-RFP intensity. Itemized in this figure is also a description of the subcellular localization at rest and during phagocytosis of each RhoGAP in the collection. A score ranging from 0 to 6 (seventh column) was assigned to members of the RhoGAP family according to the robustness of their recruitment to the phagocytic cup. The background of each entry was colour-coded to facilitate interpretation: a red background corresponds to proteins that were absent from (score = 0) or barely detectable at (score = 1) the phagocytic cup, while a green background corresponds to proteins that were moderately recruited (score = 2-4). Blue background was assigned to proteins showing moderate-to-exceptional (score = 5) or exceptional (score = 6) translocation to sites of phagocytosis. Only RhoGAPs receiving scores ranging from 4-6 were selected for further screening and functional validation.

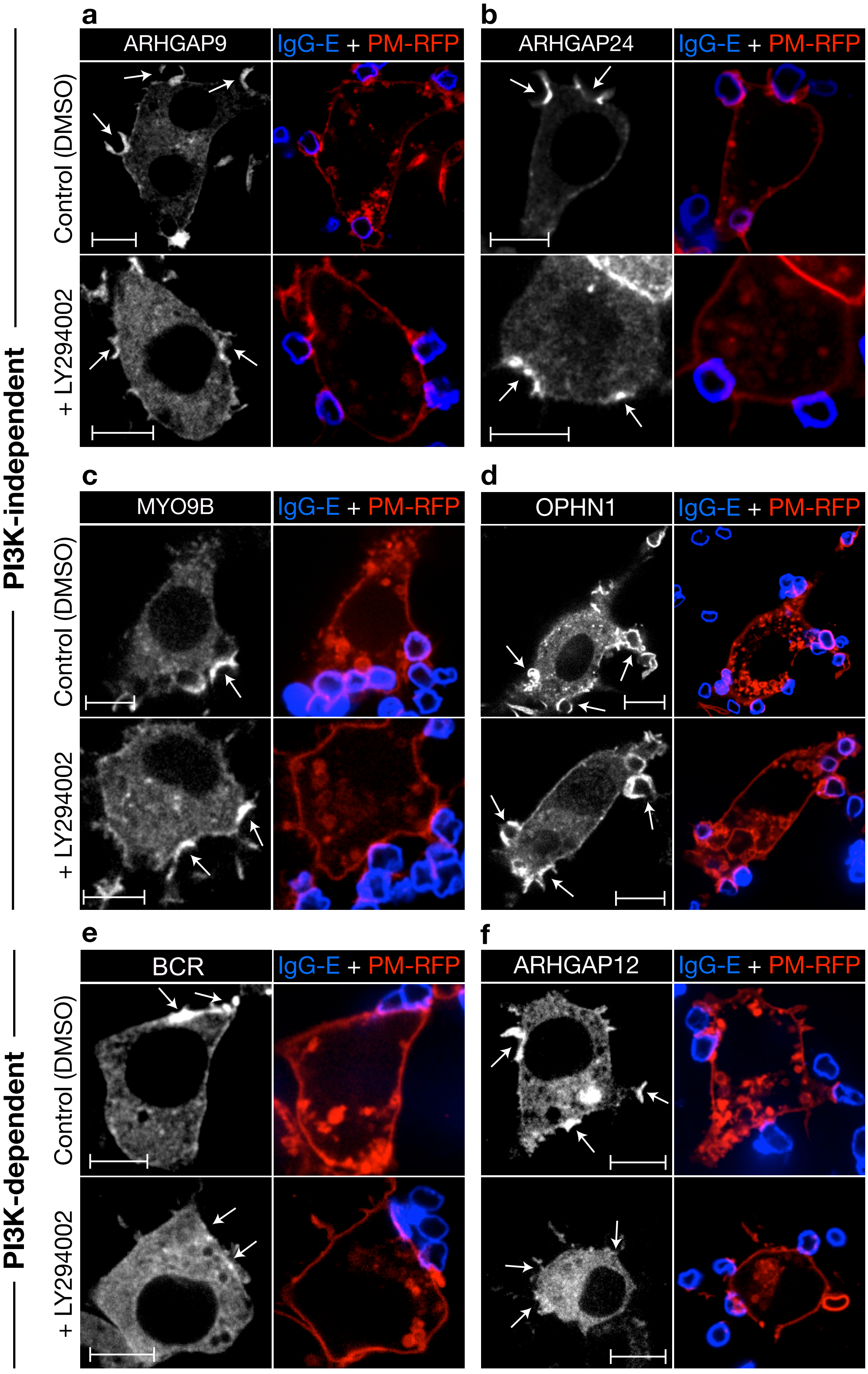

PI3K-independent
PI3K-dependent
a
ARHGAP9
IgG-E + PM-RFP
b
ARHGAP24
IgG-E + PM-RFP
c
MYO9B
IgG-E + PM-RFP
d
OPHN1
IgG-E + PM-RFP
e
BCR
IgG-E + PM-RFP
f
ARHGAP12
IgG-E + PM-RFP
Control (DMSO)
+ LY294002

Supplementary Figure 3: PI3K dependency of RhoGAP recruitment to phagocytic cups. (**a-f**) RAW 264.7 macrophages were independently co-transfected with constructs encoding the plasmalemmal marker PM-RFP and each of the 10 RhoGAPs that most markedly accumulated at phagocytic cups. Transfectants were exposed to IgG-opsonized erythrocytes (IgG-E; shown in blue) for 3 min before being fixed and imaged by confocal microscopy. Where indicated, cells were treated with either vehicle (DMSO) or the PI3K inhibitor LY294002 for 10 min prior to the addition of phagocytic targets. RhoGAPs that were recruited to sites of phagocytosis in a PI3K-independent manner are shown in the upper four panels (**a-d**), while those that were sensitive to PI3K inhibition are shown in the lower two panels (**e,f**). Arrows point to sites of particle engagement. Scale bar, 10 μm. Micrographs are representative of 3 independent experiments; at least 20 cells were assessed per replicate.

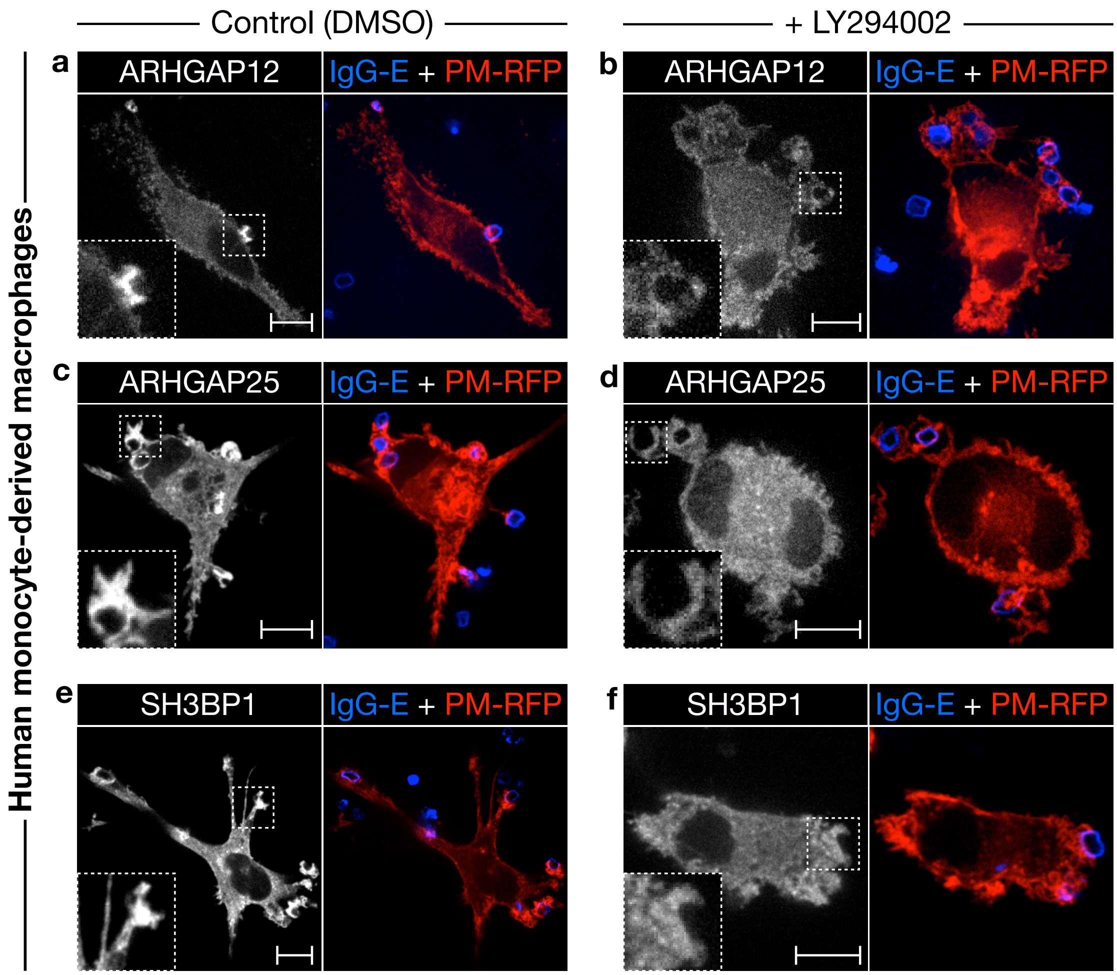

Supplementary Figure 4: ARHGAP12, ARHGAP25 and SH3BP1 translocate to phagocytic cups in a PI3K-dependent manner in primary macrophages. (**a**-**f**) Human monocyte-derived macrophages were co-electroporated with constructs encoding mCitrine-tagged ARHGAP12 (**a**,**b**), ARHGAP25 (**c**,**d**) or SH3BP1 (**e**,**f**) and the plasmalemmal marker PM-RFP. The electroporated cells were treated with either vehicle (DMSO; left panels) or the PI3K inhibitor LY294002 (right panels) for 10 min prior to being challenged with IgG-opsonized erythrocytes (IgG-E; shown in blue). Phagocytosis proceeded for 3 min before fixation and imaging by confocal microscopy. Insets (boxed regions) show magnified views of the phagocytic cup. Scale bar, 10 µm. Micrographs are representative of 3 independent experiments; at least 20 cells were assessed per replicate.

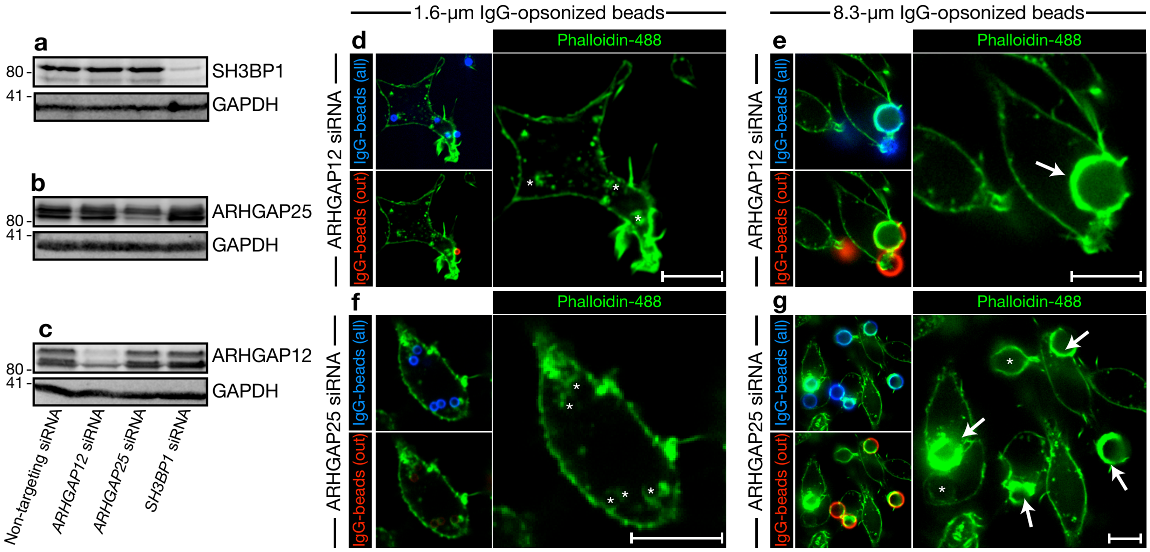

Supplementary Figure 5: Silencing of PI3K-sensitive RhoGAPs precludes phagocytosis of large, but not small phagocytic targets. (**a-c**) Representative immunoblots from 3 experiments showing protein levels of SH3BP1 (**a**), ARHGAP25 (**b**) and ARHGAP12 (**c**) in cells electroporated with the indicated siRNAs. **(d-g)** RAW 264.7 macrophages electroporated with siRNA directed against *ARHGAP12* (**d,e**) or *ARHGAP25* (**f,g**) were challenged with small (left panels) or large (right panels) IgG-opsonized phagocytic particles and fixed 10 min after particle exposure. All targets were stained with a Cy5-conjugated anti-IgG secondary antibody (shown in blue) prior to being added to phagocytes. Particles that remained extracellular (shown in red) were identified by the procedure described in the legend of Figure 4. Macrophages were then permeabilized and actin filaments stained with phalloidin-488 (shown in green). Formed phagosomes are indicated with a star. Abortive phagocytic cups, evident in cells receiving siRNA directed towards *ARHGAP12* or *ARHGAP25* and challenged with large particles, are signaled with arrows. Scale bar, 10 μm. Micrographs are representative of 3 independent experiments; at least 20 cells were assessed per replicate.

a Full gel for Figure 2g

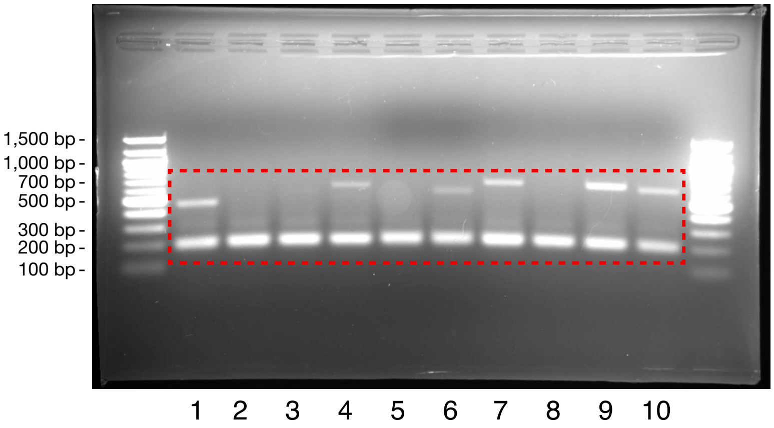

b Full blot for Supplementary Figure 5a

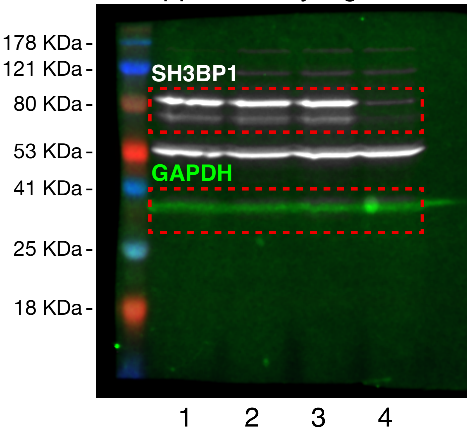

c Full blot for Supplementary Figure 5b

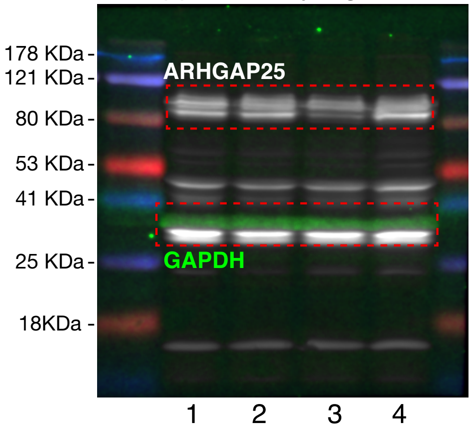

d Full blot for Supplementary Figure 5c

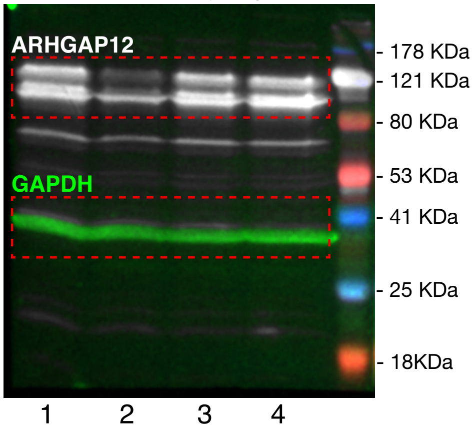

Supplementary Figure 6: Images of uncropped gels and blots. Images are shown in their original full-size form, as they appeared before being cropped for presentation purposes. Dashed red boxes demarcate the portions of the blots that are displayed in the final figures. (**a**) Full gel for figure 2g, indicating endogenous levels of expression of different RhoGAPs, as assessed by RT-PCR. The lower bands at 200 bp correspond to the positive control *GAPDH*; all other bands correspond to RhoGAP messages. The lanes in this gel are indicative of expression of the following transcripts: 1) *MYO9B*; 2) *ARHGAP24*; 3) *OPHN1*; 4) *ARHGAP25*; 5) *BCR*; 6) *ARHGAP12*; 7) *SH3BP1*; 8) *ARHGAP9*; 9) *PIK3R1* and; 10) *GRLF1*. (**b-d**) Full blots for Supplementary Fig. 5a-c. Each blot shows the relative protein levels of SH3BP1 (**b**), ARHGAP25 (**c**) or ARHGAP12 (**d**) following electroporation with a non-targeting siRNA (lane 1), or with siRNA directed against *ARHGAP12* (lane 2), *ARHGAP25* (lane 3) or *SH3BP1* (lane 4). GAPDH, detected with a fluorescently conjugated secondary antibody and shown in green, was used as a loading control in all three blots.

Name	Sequence (5'→3')	Location on target	Length (bp)	Melting Temp. (°C)	Amplicon length (bp)
Myo9B (forward)<br>*Myo9B* (reverse)	agtgtatcgtcatctctggtgagagtgg<br>catctccatggcctgctgaagc	Exon 2<br>Exon 6	28<br>22	59.9<br>60.5	473
Arhgap24 (forward)<br>*Arhgap24* (reverse)	tggaagactgggtgaagtccatcc<br>ttctgtgcgctcatcttgttaactcc	Exon 4<br>Exon 8	24<br>26	60<br>59.7	523
Ophn1 (forward)<br>*Ophn1* (reverse)	ggagttcagcgactgctacctcg<br>ttgaacttctttgactcctggacttcc	Exon 1<br>Exon 7	23<br>27	59.1<br>59.2	567
Arhgap25 (forward)<br>*Arhgap25* (reverse)	aaggtgggctggctgaagaagc<br>caggaacccttcatactgactccaagg	Exon 2<br>Exon 6	22<br>27	60.5<br>61.1	627
Bcr (forward)<br>*Bcr* (reverse)	gctgaggaacagcgccgg<br>cgtgatctcctcgttgatgctgg	Exon 2<br>Exon 8	18<br>23	58.9<br>60.4	744
Arhgap12 (forward)<br>*Arhgap12* (reverse)	gactcacattctcctaaagtttccagcc<br>gttcatccagctcttctgaccaacc	Exon 3<br>Exon 5	28<br>25	59.8<br>59.1	562
Sh3bp1 (forward)<br>*Sh3bp1* (reverse)	atcctagccgagtttgagatgaccc<br>tctgaggccatggtctgcttaagg	Exon 5<br>Exon 11	25<br>24	59.7<br>60.4	659
Arhgap9 (forward)<br>*Arhgap9* (reverse)	catggcttgctctctctgaacagc<br>tccaattcgtcatcctcaccagc	Exon 3<br>Exon 8	24<br>23	59.2<br>59.8	612
Pik3r1 (forward)<br>*Pik3r1* (reverse)	aactccgagacactgctgatggg<br>catgcgtttgtcgatctctcgg	Exon 10<br>Exon 14	23<br>22	59.1<br>59.2	653
Arhgap35 (forward)<br>*Arhgap35* (reverse)	tctgacagtgagatggacacaagttcc<br>caatctgcatgctgtatggtaccagg	Exon 2<br>Exon 5	27<br>26	59.5<br>60.5	619
Gapdh (forward)<br>*Gapdh* (reverse)	aacgggaagcccatcaccatc<br>tggttcacacccatcacaaacatgg	Exon 3<br>Exon 4	21<br>25	62.8<br>63.9	203

Supplementary Table 1: Primers employed to detect endogenous RhoGAP expression in RAW 264.7 cells.
